# Whole-Brain Mapping of Direct Inputs to Dopamine D1 and D2 Receptor-Expressing Medium Spiny Neurons in the Posterior Dorsomedial Striatum

**DOI:** 10.1523/ENEURO.0348-20.2020

**Published:** 2021-01-12

**Authors:** Jiayi Lu, Yifeng Cheng, Xueyi Xie, Kayla Woodson, Jordan Bonifacio, Emily Disney, Britton Barbee, Xuehua Wang, Mariam Zaidi, Jun Wang

**Affiliations:** Department of Neuroscience and Experimental Therapeutics, College of Medicine, Texas A&M University Health Science Center, Bryan, TX 77807

**Keywords:** BNST, CeA, dopamine D1 and D2 receptors, posterior dorsomedial striatum, rabies virus-mediated retrograde monosynaptic tracing, whole-brain mapping

## Abstract

The posterior dorsomedial striatum (pDMS) is mainly composed of medium spiny neurons (MSNs) expressing either dopamine D1 receptors (D1Rs) or D2Rs. Activation of these two MSN types produces opposing effects on addictive behaviors. However, it remains unclear whether pDMS D1-MSNs or D2-MSNs receive afferent inputs from different brain regions or whether the extrastriatal afferents express distinct dopamine receptors. To assess whether these afferents also contained D1Rs or D2Rs, we generated double transgenic mice, in which D1R-expressing and D2R-expressing neurons were fluorescently labeled. We used rabies virus-mediated retrograde tracing in these mice to perform whole-brain mapping of direct inputs to D1-MSNs or D2-MSNs in the pDMS. We found that D1-MSNs preferentially received inputs from the secondary motor, secondary visual, and cingulate cortices, whereas D2-MSNs received inputs from the primary motor and primary sensory cortices, and the thalamus. We also discovered that the bed nucleus of the stria terminalis (BNST) and the central nucleus of the amygdala (CeA) contained abundant D2R-expressing, but few D1R-expressing, neurons in a triple transgenic mouse model. Remarkably, although limited D1R or D2R expression was observed in extrastriatal neurons that projected to D1-MSNs or D2-MSNs, we found that cortical structures preferentially contained D1R-expressing neurons that projected to D1-MSNs or D2-MSNs, while the thalamus, substantia nigra pars compacta (SNc), and BNST had more D2R-expressing cells that projected to D2-MSNs. Taken together, these findings provide a foundation for future understanding of the pDMS circuit and its role in action selection and reward-based behaviors.

## Significance Statement

The dorsomedial striatum (DMS) is a brain region that has critical roles in drug addiction. The DMS receives and integrates information from multiple other regions, such as the cortex and thalamus. These extrastriatal inputs onto two DMS cell types, D1-neurons and D2-neurons, provide a substrate for how the brain segregates information process, as D1-neurons positively and D2-neurons negatively control addiction-associated behaviors. The present study found that the cortex and other brain regions that are involved in motivational and decision-making behaviors preferentially targeted D1-neurons, whereas the thalamus, which plays essential roles in behavioral flexibility, preferentially projected to D2-neurons. These findings of distinct innervations of D1-neurons and D2-neurons provide a foundation for future studies of DMS function.

## Introduction

The basal ganglia has critical roles in movement control and action selection (Balleine et al., 2009). The striatum, which provides the primary input to the basal ganglia, receives and integrates information from cortical, thalamic, and limbic structures before passing this to the basal ganglia for an appropriate action (Bamford et al., 2018). Growing evidence indicates that the dorsomedial striatum (DMS) is involved in the control of goal-directed actions and drug-seeking behaviors (Volkow et al., 2006; Wang et al., 2010; Ma et al., 2018). Two distinct subtypes of medium spiny neuron (MSN) make up as much as 95% of neurons within the DMS (Gerfen and Surmeier, 2011; Cheng et al., 2017). One type of MSN expresses dopamine D1 receptors (D1Rs) and projects to the substantia nigra pars reticulata (SNr), forming the direct pathway (Kreitzer and Malenka, 2008; Gerfen and Surmeier, 2011). The other type of MSN expresses dopamine D2 receptors (D2Rs) and projects to the external globus pallidus (GPe), forming the indirect pathway (Kreitzer and Malenka, 2008; Gerfen and Surmeier, 2011). Activation of D1R-expressing MSNs (D1-MSNs) or D2R-expressing MSNs (D2-MSNs) produces opposing effects on movement and reward-related behaviors (Gerfen et al., 2013; Cheng et al., 2017; Lüscher et al., 2020). Thus, many learned responses require the coordinated activation of the direct pathway and inhibition of the indirect pathway in the DMS. Importantly, recent studies have found that the posterior region of the DMS (pDMS) is likely to play a greater role in goal-directed behavior than the anterior DMS (aDMS; Yin and Knowlton, 2004; Yin et al., 2005b; Regier et al., 2015). The pDMS is necessary for the integration of action-outcome associations and expression of goal-directed learning (Yin and Knowlton, 2004; Yin et al., 2005a,b; Corbit and Janak, 2010; Shiflett et al., 2010). Additionally, a recent anatomic study also found different cortical inputs to the aDMS and pDMS (Hunnicutt et al., 2016). While the aDMS mainly receives inputs from the orbital, prelimbic, infralimbic, and anterior cingulate cortices, the pDMS primarily receives inputs from the motor, orbital, cingulate, and visual cortices (Hunnicutt et al., 2016). While the innervation of D1-MSNs and D2-MSNs in the dorsal striatum has been studied using the rabies virus-mediated retrograde monosynaptic tracing system (Wall et al., 2013), it remains unclear whether D1-MSNs and D2-MSNs in the pDMS receive distinct inputs from other brain areas.

In addition to receiving excitatory glutamatergic inputs from the cortex, thalamus, and amygdala, DMS D1-MSNs and D2-MSNs are modulated by dopaminergic inputs from the substantia nigra pars compacta (SNc; Bamford et al., 2018). Dopamine can alter MSN activity by acting on their postsynaptic D1Rs or D2Rs (Gerfen and Surmeier, 2011; Cheng et al., 2017; Lüscher et al., 2020) or by acting on presynaptic receptors (Wang and Pickel, 2002; Dumartin et al., 2007; Lu et al., 2019). Electron microscopy studies have provided evidence that cortical fibers in the dorsal striatum contain both D1Rs and D2Rs (Wang and Pickel, 2002; Dumartin et al., 2007), although D1R immunoreactivity was observed less frequently (Dumartin et al., 2007). A recent study also showed that cortical neurons that projected to the DMS preferentially expressed D2Rs, rather than D1Rs (Lu et al., 2019). However, these studies could not determine which specific DMS neuronal types received the D1R-expressing or D2R-expressing inputs.

In this study, we assessed D1R and D2R expression patterns in extrastriatal neurons by generating cell-type-specific Cre-expressing double transgenic mice, in which D1R-expressing and D2R-expressing cells were labeled by a fluorescent protein. To map direct inputs to D1-MSNs or D2-MSNs, we used the rabies virus-mediated retrograde tracing approach; this allowed us to label neurons that projected monosynaptically to D1-MSNs or D2-MSNs (Wall et al., 2013; Ogawa and Watabe-Uchida, 2018). We discovered that neurons in the orbital frontal, secondary motor, visual, and cingulate cortices preferentially targeted pDMS D1-MSNs. In contrast, neurons in the thalamus, primary motor cortex, and primary sensory cortex preferentially projected to pDMS D2-MSNs. Furthermore, our triple transgenic mouse models showed that the bed nucleus of the stria terminalis (BNST) and the central nucleus of the amygdala (CeA) contained abundant D2R-expressing, but few D1R-expressing, neurons. Lastly, we found that while the number of D1R-expressing or D2R-expressing neurons that projected to pDMS MSNs was low, they exhibited distinct distribution across different brain regions. These findings lay a foundation for an improved understanding of how the pDMS organizes information from multiple upstream brain regions to determine an action.

## Materials and Methods

### Reagents

Two Cre-dependent (Flex) adeno-associated virus (AAV) serotype eight vectors were employed in this study; one expressed rabies glycoprotein (RG; AAV8-Flex-RG) and the other expressed an avian membrane EnvA receptor protein (TVA) and mCherry (AV8-Flex-TVA-mCherry). These helper viruses were purchased from the University of North Carolina Vector Core. The pseudotyped rabies viruses, EnvA-SADΔG-mCherry and EnvA-SADΔG-GFP (2.04 × 10^8^ TU/ml), were obtained from the Salk Institute Vector Core. All other reagents were purchased from Sigma.

### Animals

Drd1a-Cre (D1-Cre) and Drd2-Cre (D2-Cre) mice were acquired from the Mutant Mouse Regional Resource Center. Snap25 mice and D1-tdTomato mice were purchased from The Jackson Laboratory. D1-Cre or D2-Cre mice were crossed with Snap25 mice on a C57BL/6J background to produce D1-Cre;Snap25 or D2-Cre;Snap25 offspring. D2-Cre mice were also crossed with D1-tdTomato on a C57BL/6J background to produce D1-tdTomato;D2-Cre offspring. Mouse genotypes were determined by PCR analysis of Cre or the fluorescent protein gene in tail DNA (Cre for D1-Cre and D2-Cre mice; tdT for D1-tdTomato; GFP for Snap25 mice; Wang et al., 2011, 2015; Cheng et al., 2017, 2018; Ma et al., 2018; Wei et al., 2018; Lu et al., 2019). Mice were group-housed at 23°C with a 12/12 h light/dark cycle (lights on at 11 P.M.). Food and water were provided *ad libitum*. Male two- to three-month-old mice were used in this study. All animal care procedures and experimental protocols were approved by the Institutional Animal Care and Use Committee and were performed in agreement with the National Research Council Guide for the Care and Use of Laboratory Animals.

### Stereotaxic virus infusion

Stereotaxic viral infusions were performed as described previously (Wang et al., 2015; Cheng et al., 2017; Huang et al., 2017; Ma et al., 2017, 2018; Lu et al., 2019; Roltsch Hellard et al., 2019). Briefly, mice were anesthetized using isoflurane and mounted in a rodent stereotaxic frame (Kopf). The skin was opened to uncover the skull and expose bregma, lamda, and the location of the desired injection site. A three-axis micromanipulator was used to measure the spatial coordinates for bregma and lamda. Small drill holes were made in the skull at the appropriate coordinates, according to the Paxinos atlas (Franklin and Paxinos, 2007). Two microinjectors were loaded with 0.5 μL of a 1:1 mixture of AAV8-Flex-RG and AAV8-Flex-TVA-mCherry and then lowered into the pDMS (AP: 0.0 mm, ML: ±1.87 mm, DV: −2.90 mm). This helper virus mixture was infused into the brain at a rate of 0.1 μl/min. To avoid the backflow of the virus, microinjectors were left in place for 10 min after the infusion was complete and were then removed. The skin was sutured, and the mice were allowed to recover for three weeks before the infusion of pseudotyped rabies virus (EnvA-SADΔG-mCherry or EnvA-SADΔG-eGFP). The rabies virus was injected at the same site and using the same injection volume as the initial helper virus injection. To prevent coincident rabies infection along the AAV injection tract, the rabies virus was infused at an angle of 10° (Wall et al., 2013) into adapted coordinates (AP, 0.0 mm; ML, ± 2.42 mm; DV, −2.94 mm). The modified coordinates were calculated by measuring from the midline and parallel to the dorsal-ventral axis.

### Confocal imaging and cell counting

Rabies virus was allowed to replicate and spread for 7 d before perfusing the mice intracardially with 4% paraformaldehyde (PFA) in PBS (Wall et al., 2013). The brains were extracted and postfixed overnight in 4% PFA/PBS solution, followed by dehydration in 30% sucrose. Each whole brain was sectioned serially into 50-μm coronal sections using a cryostat. We mounted sections in a one in four series. Confocal images were obtained using a confocal laser-scanning microscope (Fluoview 1200, Olympus). Fluorescent images were reconstructed in three dimensions, and cell counts from these scans were manually acquired using the Bitplane Imaris 8.3.1 (Bitplane), as previously reported (Wei et al., 2018; Lu et al., 2019). Green and red neurons were counted using the Spot module within Imaris, which also calculated colocalization. Brain structures were registered using the Paxinos mouse atlas as a reference (Franklin and Paxinos, 2007).

### Statistical analysis

Data were analyzed by two-tailed *t* test, two-way ANOVA with repeated measurement (RM), or one-way ANOVA followed by the Tukey’s *post hoc* test. Significance was determined if *p *<* *0.05. Statistical analysis was conducted by SigmaPlot.

## Results

### Identification of distinct whole-brain extrastriatal inputs to D1-MSNs and D2-MSNs in the pDMS

**Figure 1.**
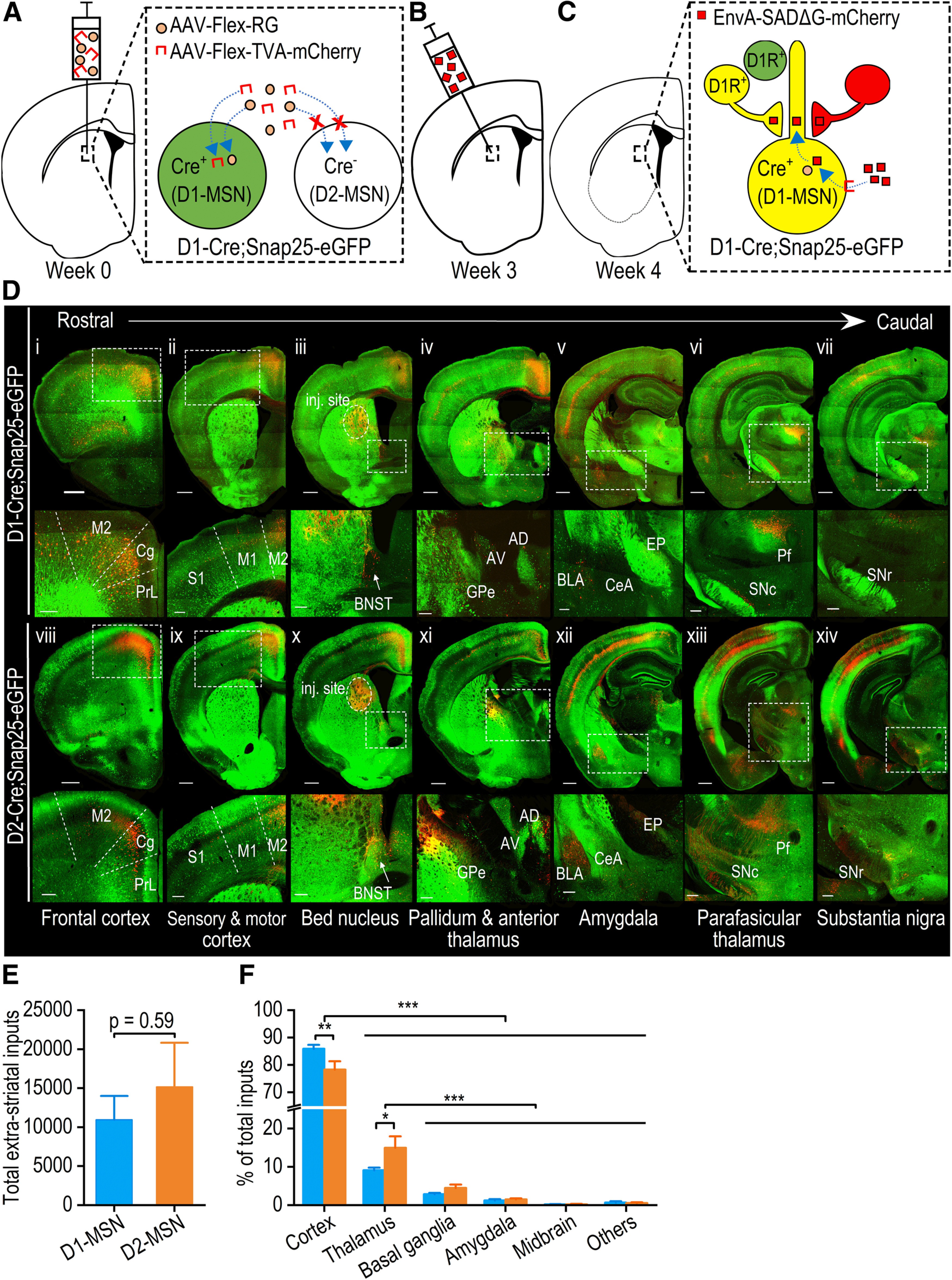
Rabies virus-mediated retrograde monosynaptic whole-brain tracing of neurons projecting to D1-MSNs and D2-MSNs in the pDMS. ***A***, ***B***, The experimental design used to trace neurons with afferent inputs to D1-MSNs in the pDMS. The design shows how we employed D1-Cre;Snap25 transgenic mice, in which D1-MSNs selectively expressed Cre and GFP (green). Note that the same approach was repeated in D2-Cre;Snap25 and D1-tdTomato;D2Cre mice, where we traced afferent inputs to D2-MSNs. Selective rabies infection of D1-MSNs was achieved by injecting the pDMS of these mice with Cre-dependent helper viruses expressing an avian membrane EnvA receptor protein (TVA) and RG (AAV-Flex-TVA-mCherry and AAV-Flex-RG) three weeks before injection of a modified rabies virus (EnvA-SADΔG-mCherry) into the same area at a 10° angle. ***C*,** One week after rabies injection, the rabies virus had specifically infected TVA-expressing D1-MSNs and spread retrogradely from RG-expressing D1-MSNs to neurons with monosynaptic connections with them. Note that extrastriatal neurons with monosynaptic connections to RG-expressing D1-MSNs expressed rabies-derived mCherry (red). mCherry-expressing neurons that also contained D1Rs and thus expressed Cre-driven GFP (green) appeared yellow. Extrastriatal neurons that expressed D1Rs but did not make any connections with RG-expressing D1-MSNs were labeled green. ***D*,** Representative confocal images of rabies virus-labeled mCherry-expressing neurons (red) that project to D1-MSNs (i–vii) or D2-MSNs (viii–xiv) throughout the brain in the D1-Cre;Snap25 or D2-Cre;Snap25 mice, respectively. Rows 2 and 4 show enlarged images of the boxed areas in rows 1 and 3, respectively. Note that there were extensive mCherry-positive neurons in the cortex (i–xiv), BNST (iii, x), GPe (iv, xi), amygdala (v, xii), thalamus (vi, xiii), and midbrain (vii, xiv). M2, secondary motor cortex; Cg, cingulate cortex; PrL, prelimbic cortex; S1, primary sensory cortex; M1, primary motor cortex; BNST, bed nucleus of the stria terminalis; GPe, globus pallidus external; AD, anterior dorsal thalamus; AV, anterior ventral thalamus; BLA, basolateral amygdala; CeA, central amygdala; EP, entopeduncular nucleus; Pf, parafascicular thalamic nucleus; SNc, substantia nigra pars compacta; SNr, substantia nigra pars reticulata; inj. site, injection site. Scale bars: 500 μm (rows 1 and 3) and 200 μm (rows 2 and 4). ***E*,** There was no significant difference between the total number of extrastriatal neurons with projections to D1-MSNs or D2-MSNs; unpaired *t* test. ***F*,** Extrastriatal inputs onto D1-MSNs (blue) versus D2-MSNs (orange); *p <* *0.05, **p <* *0.01, ***p <* *0.001; two-way RM ANOVA followed by Tukey’s test for the indicated comparisons; *n* = 5 mice for D1-MSNs (D1-Cre;Snap25 mice) and *n* = 8 mice for D2-MSNs (4 D2-Cre;Snap25 mice and 4 D1-tdTomato;D2-Cre mice; ***E***, ***F***).

To compare afferent inputs onto pDMS D1-MSNs and D2-MSNs, we employed rabies-mediated monosynaptic retrograde tracing in D1-Cre;Snap25 and D2-Cre;Snap25 mice, in which D1-MSNs or D2-MSNs selectively expressed Cre (and thus GFP), respectively (Madisen et al., 2015; Wei et al., 2018; Lu et al., 2019). In these mouse lines, the pDMS was injected with Cre-dependent AAVs that expressed TVA-mCherry and RG (Fig. 1*A*). TVA facilitated neuronal infection by the pseudotyped rabies virus, while RG facilitated retrograde monosynaptic spread of the rabies virus from infected neurons (Wall et al., 2013; Ogawa and Watabe-Uchida, 2018). Three weeks after the infusion of these vectors, we injected the glycoprotein-deleted pseudotyped rabies virus, EnvA-SADΔG-mCherry, at the same pDMS location using an angled injection tract (Fig. 1*B*) to prevent coincident infection (Wall et al., 2013). This strategy of selectively expressing TVA and RG in Cre-expressing D1-MSNs in D1-Cre;Snap25 mice or D2-MSNs in the D2-Cre;Snap25 mice meant that the rabies virus selectively infected these neurons and spread to neurons with monosynaptic inputs to them (Fig. 1*C*). One week after the rabies virus infusions, serial coronal 50-μm sections of the whole brain were prepared, and every fourth section was imaged using confocal laser-scanning microscopy. We observed intense GFP expression in the striatum and at D1-MSN projection targets, including the GPe (Fig. 1*Div*), entopeduncular nucleus (Fig. 1*Dv*), and SNr (Fig. 1*Dvii*), and at D2-MSN projection targets such as the GPe (Fig. 1*Dxi*). We also observed a large number of mCherry-positive extrastriatal neurons with monosynaptic connections to D1-MSNs (Fig. 1*Di-Dvii*) or D2-MSNs (Fig. 1*Dviii-Dxiv*); these were located in the cortex (Fig. 1*Di-Dxiv*), BNST (Fig. 1*Diii, Dx*), GPe (Fig. 1*Div, Dxi*), amygdala (Fig. 1*Dv, Dxii*), thalamus (Fig. 1*Dvi, Dxiii*), and the midbrain (Fig. 1*Dvii*, *Dxiv*).

These rabies-labeled afferent (mCherry-positive) neurons in both hemispheres were counted relative to brain region boundaries. The total number of extrastriatal mCherry-positive neurons did not differ significantly between D1-Cre;Snap25 and D2-Cre;Snap25 mice (*t*_(11)_ = −0.55, *p *>* *0.05; Fig. 1*E*). Since both AAV8-Flex-TVA-mCherry and the rabies virus expressed mCherry, this did not allow for selective visualization of starter cells, which were infected by both the rabies virus and AAV8-Flex-RG. Thus, labeled neuron counts from any given brain region were normalized to the total inputs to the pDMS detected within each animal. This approach was used to account for any inter-animal differences in viral infusion. This analysis identified significant differences in the extent of inputs from distinct brain regions (*F*_(5,55)_ = 627.19, *p *<* *0.001; Fig. 1*F*). Specifically, the majority of inputs onto pDMS D1-MSNs and D2-MSNs arose from the cortex (*p *<* *0.001 for cortex vs thalamus, basal ganglia, amygdala, midbrain, and others; Fig. 1*F*), and from the thalamus (*p *<* *0.001 for thalamus vs basal ganglia, amygdala, midbrain, and others; Fig. 1*F*). Interestingly, cortical neurons preferentially innervated D1-MSNs versus D2-MSNs, whereas thalamic neurons preferentially connected with D2-MSNs versus D1-MSNs (cortical neurons: *p *<* *0.01; thalamic neurons: *p *<* *0.05; Fig. 1*F*).

**Figure 2.**
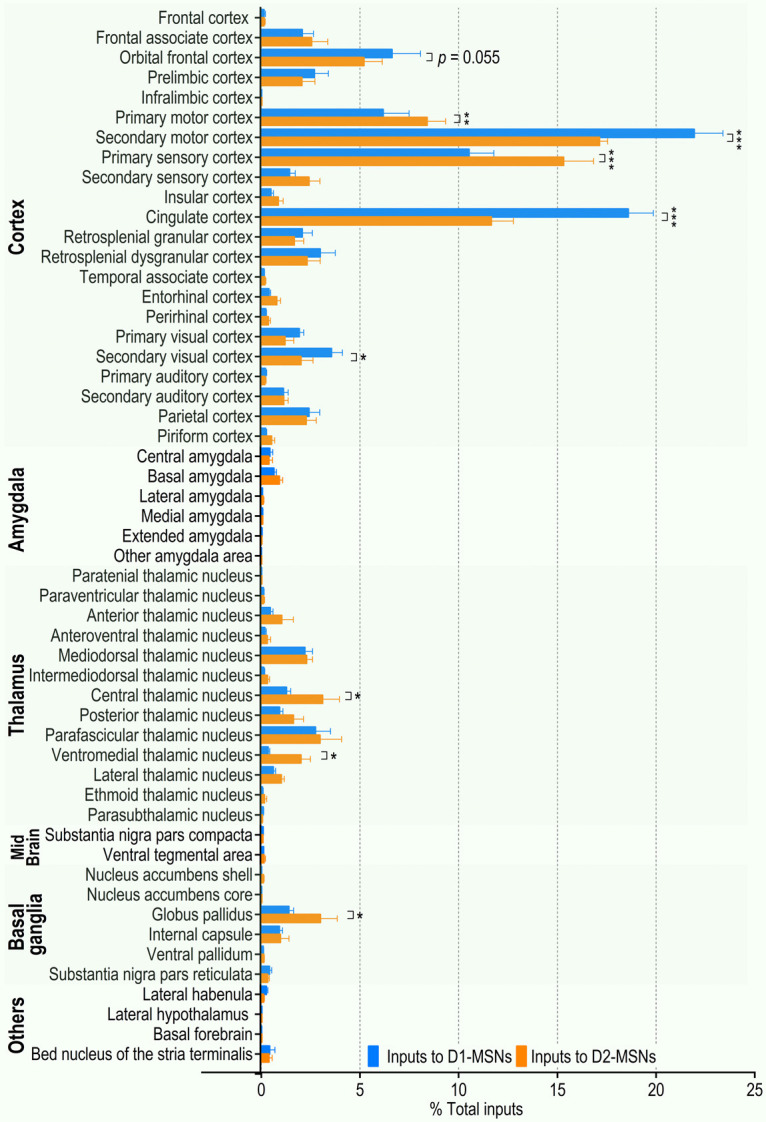
Summary of brain-wide monosynaptic inputs to pDMS D1-MSNs and D2-MSNs. The majority of direct synaptic inputs to the pDMS arose in the cortex and thalamus. Analysis of the normalized distribution of rabies virus-labeled neurons showed that the orbital frontal cortex, secondary motor cortex, cingulate cortex, and secondary visual cortex preferentially projected to D1-MSNs versus D2-MSNs. The primary motor cortex, primary sensory cortex, central thalamic nucleus, ventromedial thalamic nucleus, and globus pallidus preferentially innervated D2-MSNs versus D1-MSNs. The extrastriatal inputs onto D1-MSNs (blue) or D2-MSNs (orange) are expressed as a percentage of the total inputs to these cell types; *p <* *0.05, **p <* *0.01, ***p <* *0.001; two-way RM ANOVA followed by Tukey’s test; *n* = 5 mice for D1-MSNs (D1-Cre;Snap25 mice) and *n* = 8 mice for D2-MSNs (4 D2-Cre;Snap25 mice and 4 D1-tdTomato;D2-Cre mice).

Both the cortex and thalamus contain many subregions. Within the cortex, the most prominent inputs to D1-MSNs (expressed as a percentage of total inputs) were from the orbital frontal cortex (6.62 ± 1.45%), primary (6.17 ± 1.31%) and secondary (21.93 ± 1.47%) motor cortices, primary sensory cortex (10.53 ± 1.25%), and cingulate cortex (18.59 ± 1.27%; Fig. 2). For D2-MSNs, the equivalent percentages were 5.20 ± 0.93%, 8.40 ± 0.94%, 17.13 ± 0.42%, 15.31 ± 1.52%, and 11.66 ± 1.10% of total D2-MSN inputs, respectively (Fig. 2). Within the thalamus, the parafascicular nucleus, mediodorsal thalamic nucleus, and central thalamic nucleus gave rise to the majority of thalamostriatal inputs to D1-MSNs and D2-MSNs (2.75 ± 0.77%, 2.21 ± 0.40%, and 1.28 ± 0.21% of total D1-MSN inputs, respectively; and 2.97 ± 1.10%, 2.29 ± 0.31%, and 3.10 ± 0.86% of total D2-MSN inputs, respectively; Fig. 2). These data suggest that pDMS MSNs receive inputs primarily from the cortex and thalamus, which preferentially project to D1-MSNs and D2-MSNs, respectively.

Previous studies have demonstrated that the striatum plays a critical role in sorting sensory, motor, and reward information arriving from two major excitatory sources: the cortex and thalamus (Lovinger, 2010; Bamford et al., 2018). Differential activation of the direct and indirect pathways subsequently results in the selection and execution of appropriate responses. Therefore, we next analyzed whether there were cortical or thalamic subregions that preferentially targeted D1-MSNs or D2-MSNs. Although many cortical structures provided similar proportions of the inputs to D1-MSNs and D2-MSNs, we identified some cortical regions that showed a considerable bias in their synaptic input to a specific cell type. The secondary motor, cingulate, and secondary visual cortices provided significantly higher proportions of the inputs to D1-MSNs, as compared with D2-MSNs (*p *<* *0.001, 0.001, and * *0.05, respectively, Figs. 1*D*, 2). This trend was also observed in the orbital frontal cortex (*p *=* *0.055; Fig. 2). In contrast, the primary motor and primary sensory cortices provided more inputs to D2-MSNs than to D1-MSNs (*p *<* *0.05 and 0.001, respectively; Figs. 1*D*, 2). The other major source of glutamatergic input to the striatum, the thalamostriatal projections, also showed some biased connectivity. Both the central and ventromedial thalamic nuclei provided significantly higher proportions of inputs to D2-MSNs than to D1-MSNs (*p *<* *0.05; Figs. 1*D*, 2). In addition to these cortical and thalamic inputs, the amygdala provides glutamatergic inputs to the DMS. The present study found similar proportions of synaptic inputs to D1-MSNs and D2-MSNs from the central, basal, and lateral amygdala (*p *>* *0.05 for all areas; Figs. 1*D*, 2).

We also compared the projections from the SNc, a known dopaminergic input to the DMS, to D1-MSNs and D2-MSNs (Surmeier et al., 2007). Consistent with a previous report (Wall et al., 2013), we found that a relatively small proportion of the total inputs were from the SNc, and there was no significant difference between the percentages of these inputs to D1-MSNs and D2-MSNs (*p *>* *0.05; Figs. 1*D*, 2). Lastly, we compared the GABAergic inputs from the GPe to the DMS (Lee et al., 2004; Hernández et al., 2015) and found significantly greater D2-MSN innervation (*p *<* *0.05; Figs. 1*D*, 2).

Taken together, these observations demonstrated input specificity onto two MSN subtypes in the pDMS. We discovered that the secondary motor, secondary visual, and cingulate cortices preferentially targeted D1-MSNs, whereas inputs from the thalamus, primary motor cortex, and primary sensory cortex preferentially projected to D2-MSNs. Consequently, the cortex and thalamus preferentially projected to pDMS D1-MSNs and D2-MSNs, respectively.

### Preferential expression of D2Rs versus D1Rs in the BNST and CeA

**Figure 3.**
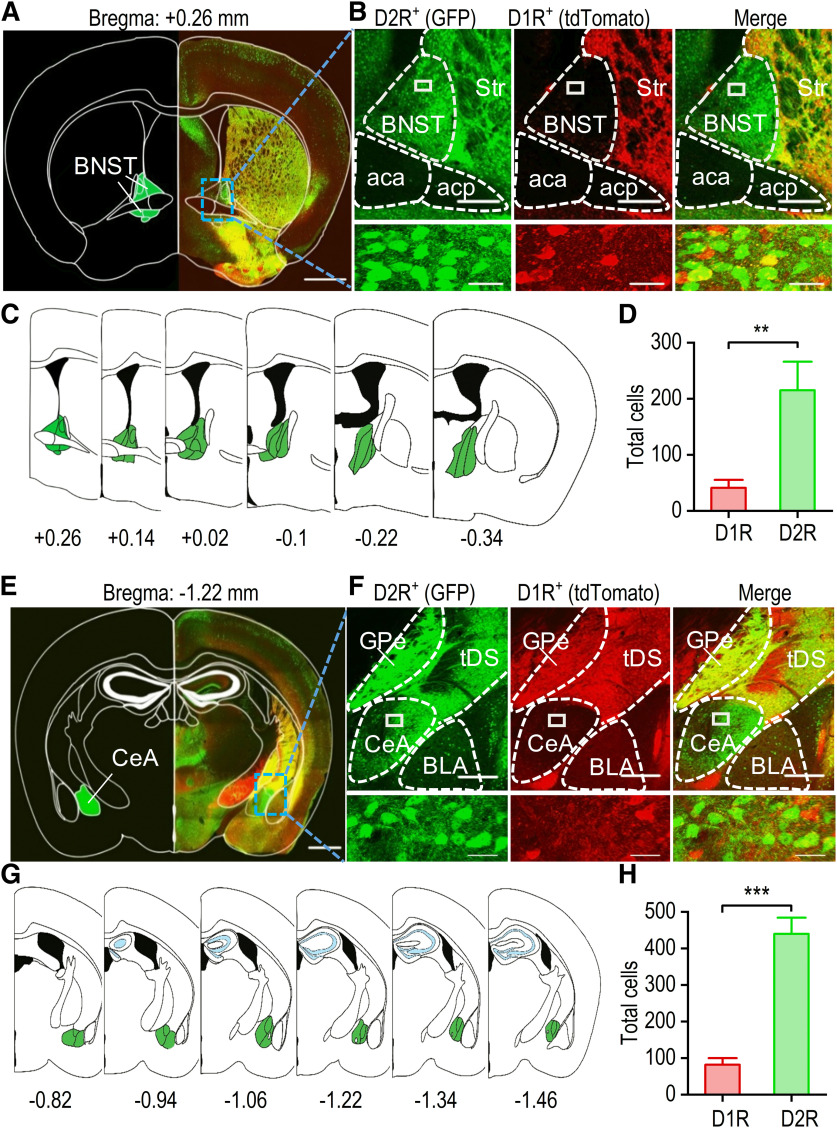
The BNST and CeA preferentially express D2Rs versus D1Rs. ***A***, Representative fluorescent image of a coronal section of the BNST from a D1-tdTomato;D2-Cre;Snap25 mouse. The atlas skeleton (left) shows the BNST location at +0.26 mm relative to bregma. ***B*,** Representative dual-channel higher magnification fluorescent images of the boxed region of panel A showing abundant GFP-expressing (D2R-positive) neurons (left), a few tdTomato-expressing (D1R-positive) neurons (middle), and some colocalization (right). The bottom panels show an enlarged image of the boxed region from the top panels. Str, striatum; aca, anterior commissure area; acp, posterior commissure area. ***C*,** Schematic representation of the BNST starting at +0.26 mm and ending at −0.34 mm relative to bregma. ***D*,** Bar graph summarizing the numbers of D1R-expressing and D2R-expressing neurons in the BNST; **p <* *0.01, unpaired *t* test; *n* = 18 sections from three mice. ***E*,** Representative fluorescent image of a coronal section of the CeA from a D1-tdTomato;D2-Cre;Snap25 mouse. The atlas skeleton (left) shows the location of the CeA at −1.22 mm relative to bregma. ***F*,** Representative dual-channel higher magnification fluorescent images of the boxed region of panel E showing abundant GFP-expressing D2R-positive neurons (left), a few tdTomato-expressing D1R-positive neurons (middle), and some colocalization (right). The bottom panels show enlarged images of the boxed region of the top panels. GPe, globus pallidus external; tDS; tail of the striatum; BLA, basolateral amygdala. ***G*,** Schematic representation of the CeA starting at −0.82 mm and ending at −1.46 mm relative to bregma. ***H*,** Bar graph depicting the higher average number of D2R-expressing neurons than D1R-expressing neurons in the CeA; ***p <* *0.001, unpaired *t* test; *n* = 18 sections from three mice. Scale bars: 1 mm (***A***, ***E***), 250 μm (***B***, ***F***, top panels), 20 μm (***B***, ***F***, bottom panels).

Having identified the distinct extrastriatal inputs to D1-MSNs versus D2-MSNs, we next asked whether there are any differences in the presynaptic inputs onto D1-MSNs or D2-MSNs in terms of their D1R and D2R expression, especially from the reward-associated brain areas that project to the DMS (e.g., cortex, amygdala, and BNST). Interestingly, although dopamine receptors are widely distributed in the brain, the receptor subtype densities vary between different areas. A previous study discovered that D1R-expressing or D2R-expressing neurons were highly segregated in the orbitofrontal cortex, prefrontal cortex, and basolateral amygdala (Wei et al., 2018). However, the distribution pattern of D1R-expressing and D2R-expressing remains unclear in the BNST and CeA. The BNST and CeA play crucial roles in many neuropsychiatric disorders that include fear formation, anxiety, or reward-related impulsivity (Davis et al., 2010; Kim et al., 2018). Dopaminergic afferents from the ventral tegmental area innervate the amygdala and BNST, but the detailed D1R-expression and D2R-expression patterns remain unclear. Therefore, we crossed D1-tdTomato transgenic mice, which expressed tdTomato under the control of the D1R gene, with D2-Cre;Snap25 mice. We counted neurons expressing D1Rs (tdTomato-positive) and D2Rs (GFP-positive) in coronal sections containing the BNST and CeA of these D1-tdTomato;D2-Cre;Snap25 transgenic mice. Strong expression of GFP and tdTomato was observed in the striatum (Fig. 3*A*,*B*). However, while strong GFP expression was present in the BNST area, tdTomato expression was barely detectable (Fig. 3*A*,*B*). For each mouse, we examined six BNST sections from bregma AP +0.26 to −0.34 mm (Fig. 3*C*). The average number of D1R-expressing neurons (41) was significantly lower than the average number of D2R-expressing neurons (215; *t*_(34)_ = −3.28, *p *<* *0.01; Fig. 3*D*).

A previous study showed relatively high D1R expression and weak D2R expression in the basolateral amygdala (Wei et al., 2018). Interestingly, using the same transgenic animals, the present study identified a high level of D2R expression in the CeA (Fig. 3*E*,*F*). We examined six CeA sections from bregma AP −0.82 to −1.46 mm (Fig. 3*G*). An average of 82 D1R-expressing neurons was observed, and this value was significantly lower than the average number of D2R-expressing neurons (439; *t*_(34)_ = −7.42, *p *<* *0.001; Fig. 3*H*). These results indicate that D2Rs are preferentially expressed in the BNST and CeA.

### Whole-brain mapping of D1R-expressing or D2R-expressing inputs to pDMS MSN subtypes

The above results (Fig. 3) and recent transgenic models have revealed a tight segregation of D1Rs and D2Rs in some reward-related structures, including the orbitofrontal cortex, prefrontal cortex, and amygdala (Wei et al., 2018). In addition to the differential innervation of D1-MSNs and D2-MSNs, it is essential to note that previous anatomic studies indicated that cortical fibers in the dorsal striatum contained abundant D2Rs, but few D1Rs (Wang and Pickel, 2002; Dumartin et al., 2007). In addition, a recent study reported that DMS-projecting extrastriatal neurons preferentially expressed D2Rs, rather than D1Rs (Lu et al., 2019). We thus asked whether extrastriatal D1R-expressing or D2R-expressing inputs showed biased projections to D1-MSNs or D2-MSNs in the pDMS. To test this, we measured the overlap between neurons that projected to D1-MSNs or D2-MSNs in the pDMS, and extrastriatal neurons that expressed D1Rs or D2Rs. To explore the anatomic connections between extrastriatal D1R-expressing inputs and D1-MSNs (D1→D1), and between D2R-expressing inputs and D2-MSNs (D2→D2), we analyzed the numbers of neurons that were both GFP-positive and tdTomato-positive in D1-Cre;Snap25 and in D2-Cre;Snap25 mice following EnvA-SADΔG-mCherry injection.

**Figure 4.**
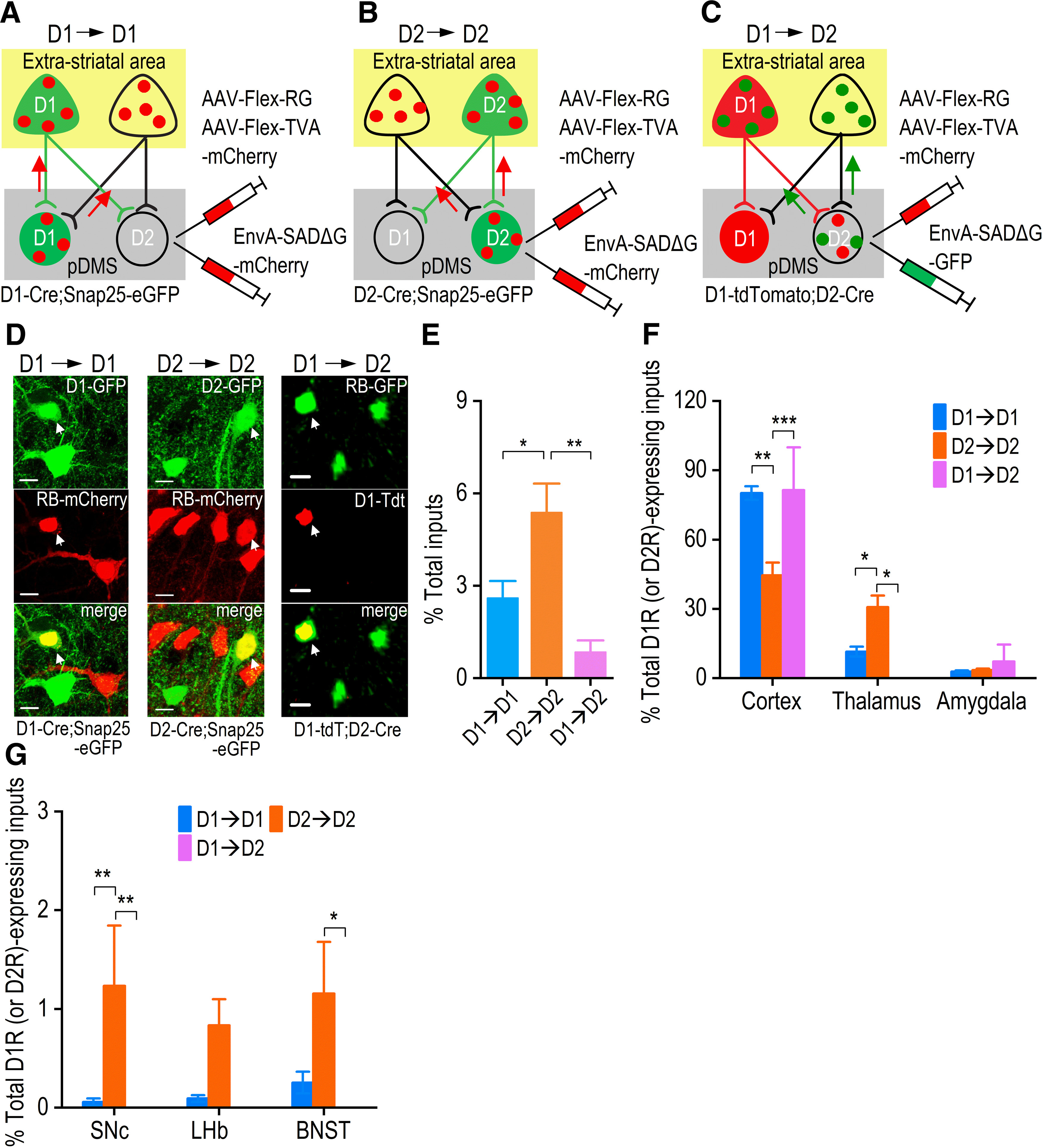
Rabies virus-mediated retrograde monosynaptic whole-brain labeling of D1R-expressing and D2R-expressing neurons projecting to D1-MSNs and D2-MSNs in the pDMS. ***A–C***, Schematic showing the experimental approach used to label extrastriatal D1R-expressing or D2R-expressing neurons with projections to pDMS D1-MSNs or D2-MSNs. D1-Cre;Snap25 mice were employed to identify D1→D1 connections (***A***), D2-Cre;Snap25 mice for D2→D2 connections (***B***), and D1-tdTomato;D2-Cre mice for D1→D2 connections (***C***). D1-Cre;Snap25 and D2-Cre;Snap25 mice expressed GFP in D1R-expressing and D2R-expressing neurons, respectively (***A***, ***B***). In D1-tdTomato;D2-Cre mice, D1R-expressing neurons were labeled red (***C***). Injection of Cre-dependent helper viruses (AAV-Flex-TVA-mCherry and AAV-Flex-RG) into the pDMS induced selective expression of TVA and RG in Cre-expressing D1-MSNs (***A***), D2-MSNs (***B***), and D2-MSNs (***C***). Three weeks after helper virus infusion, injection of EnvA-SADΔG-mCherry into the same site of the D1-Cre;Snap25 (***A***) and D2-Cre;Snap25 (***B***) mice, and of EnvA-SADΔG-GFP into the same site of D1-tdTomato;D2-Cre mice (***C***) caused selective rabies infection and expression of mCherry by D1-MSNs (***A***) or D2-MSNs (***B***), and expression of rabies-GFP by D2-MSNs (***C***). Retrograde spread of rabies virus then occurred from D1-MSNs (***A***) or D2-MSNs (***B***, ***C***) to extrastriatal presynaptic neurons. This facilitated identification of D1→D1 (***A***), D2→D2 (***B***), and D1→D2 (***C***) connections, as indicated. ***D*,** Representative confocal images of the cortex in the indicated mice following injection of either EnvA-SADΔG-mCherry or EnvA-SADΔG-GFP. White arrows indicate colocalization of D1→D1 (left), D2→D2 (middle), and D1→D2 (right). Scale bars: 10 μm. ***E*,** The relative levels of the indicated connections, where D1→D1 is expressed as a percentage of the total number of D1-MSN inputs, and D2→D2 or D1→D2 is expressed as a percentage of the total number of D2-MSN inputs; *p <* *0.05, **p <* *0.01 for the indicated comparisons, one-way ANOVA followed by Tukey’s test. ***F*,** Bar graph comparing D1R-expressing and D2R-expressing inputs from the cortex, amygdala, and thalamus onto MSNs. D1R-expressing or D2R-expressing inputs from the indicated brain regions were normalized to the total extrastriatal D1R-expressing or D2R-expressing inputs, as appropriate. Note that the cortex exhibited high percentages of D1→D1 and D1→D2 connections, whereas the thalamus showed a high percentage of D2→D2 connections; ***p <* *0.001, *p <* *0.05, two-way RM ANOVA followed by Tukey’s test. ***G*,** Bar graph comparing D1R-expressing and D2R-expressing inputs from the SNc, lateral habenula (LHb), and BNST onto MSNs. D1R-expressing or D2R-expressing inputs from these brain regions were normalized to the total extrastriatal D1R-expressing or D2R-expressing inputs; **p <* *0.01, *p <* *0.05, two-way RM ANOVA followed by Tukey’s test; *n* = 5 (D1→D1), *n* = 4 (D2→D2), *n* = 4 (D1→D2) mice (***E–G***).

D1R-expressing or D2R-expressing neurons were selectively labeled with GFP (Fig. 4*A*,*B*,*D* left, *D* middle) and the EnvA-SADΔG-mCherry rabies virus infected D1-MSNs or D2-MSNs before spreading to neurons with direct inputs to these cells (Fig. 4*A*,*B*,*D* left, *D* middle). As a result, neurons that projected to D1-MSNs or D2-MSNs were labeled red (mCherry) in these mice. To probe the connections between extrastriatal D1R-expressing inputs and D2-MSNs (D1→D2), we employed D1-tdTomato;D2-Cre mice, in which extrastriatal D1R-expressing neurons and pDMS D1-MSNs were both labeled red (tdTomato; Fig. 4*C*,*D* right). These mice did not express GFP in D2-MSNs, and GFP was subsequently used in the rabies virus. Injection of AAV8-Flex-TVA-mCherry and AAV8-Flex-RG into the pDMS, followed by EnvA-SADΔG-GFP, led to the infection of Cre-positive D2-MSNs and extrastriatal inputs to D2-MSNs by rabies virus; these expressed GFP and were thus labeled green (Fig. 4*C*,*D* right). Note that pDMS-projecting neurons that contained D1Rs (or D2Rs) showed co-expression of mCherry (or tdTomato) and GFP and were thus yellow (Fig. 4*D*).

To our surprise, the majority of extrastriatal neurons that projected to D1-MSNs or D2-MSNs did not express D1Rs or D2Rs. Specifically, the D1R was expressed by 2.6 ± 0.5% of the rabies virus-labeled neurons that projected to D1-MSNs in D1-Cre;Snap25 mice (D1→D1), and the D2R was expressed by 5.4 ± 0.9% of the inputs to D2-MSNs in D2-Cre;Snap25 mice (D2→D2). The D1R was expressed by 0.8 ± 0.4% of the inputs to D2-MSNs in D1-tdTomato;D2-Cre mice (D1→D2). We directly compared the number of D1R-expressing neurons that project to the D1-MSNs (D1→D1), D2R-expressing neurons that project to the D2-MSNs (D2→D2), and D1R-expressing neurons that project to the D2-MSNs (D1→D2), and we found that there were significantly more D2→D2 connections than either D1→D1 or D1→D2 connections (*p *<* *0.05 and *p *<* *0.01, respectively; Fig. 4*E*). This was consistent with previous anatomic studies showing greater D2R expression, as compared with D1R expression, at presynaptic terminals in the dorsal striatum (Wang and Pickel, 2002; Dumartin et al., 2007; Lu et al., 2019).

Interestingly, although the number of extrastriatal D1R-expressing or D2R-expressing neurons that projected to D1-MSNs or D2-MSNs was low, these neurons showed distinct distribution patterns across the brain. We normalized D1R-expressing or D2R-expressing inputs from individual brain regions to the total number of extrastriatal D1R-expressing or D2R-expressing inputs. Distinct D1→D1, D2→D2, and D1→D2 connections were observed in cortical and thalamic structures, but not in the amygdala (*F*_(2,20)_ = 60.22, *p *<* *0.001; Fig. 4*F*). Cortical regions had higher percentages of D1→D1 or D1→D2 connection, as compared with D2→D2 (*p *=* *0.001; Fig. 4*F*), while the thalamus had a higher percentage of D2→D2 than of D1→D1 or D1→D2 (*p *<* *0.05; Fig. 4*F*). In the SNc, a higher percentage of the connections was D2→D2, as compared with D1→D1 or D1→D2 (*p *<* *0.01; Fig. 4*G*). In the BNST, a higher percentage of the connections was D2→D2, as compared with D1→D2 (*p *<* *0.05; Fig. 4*G*).

**Figure 5.**
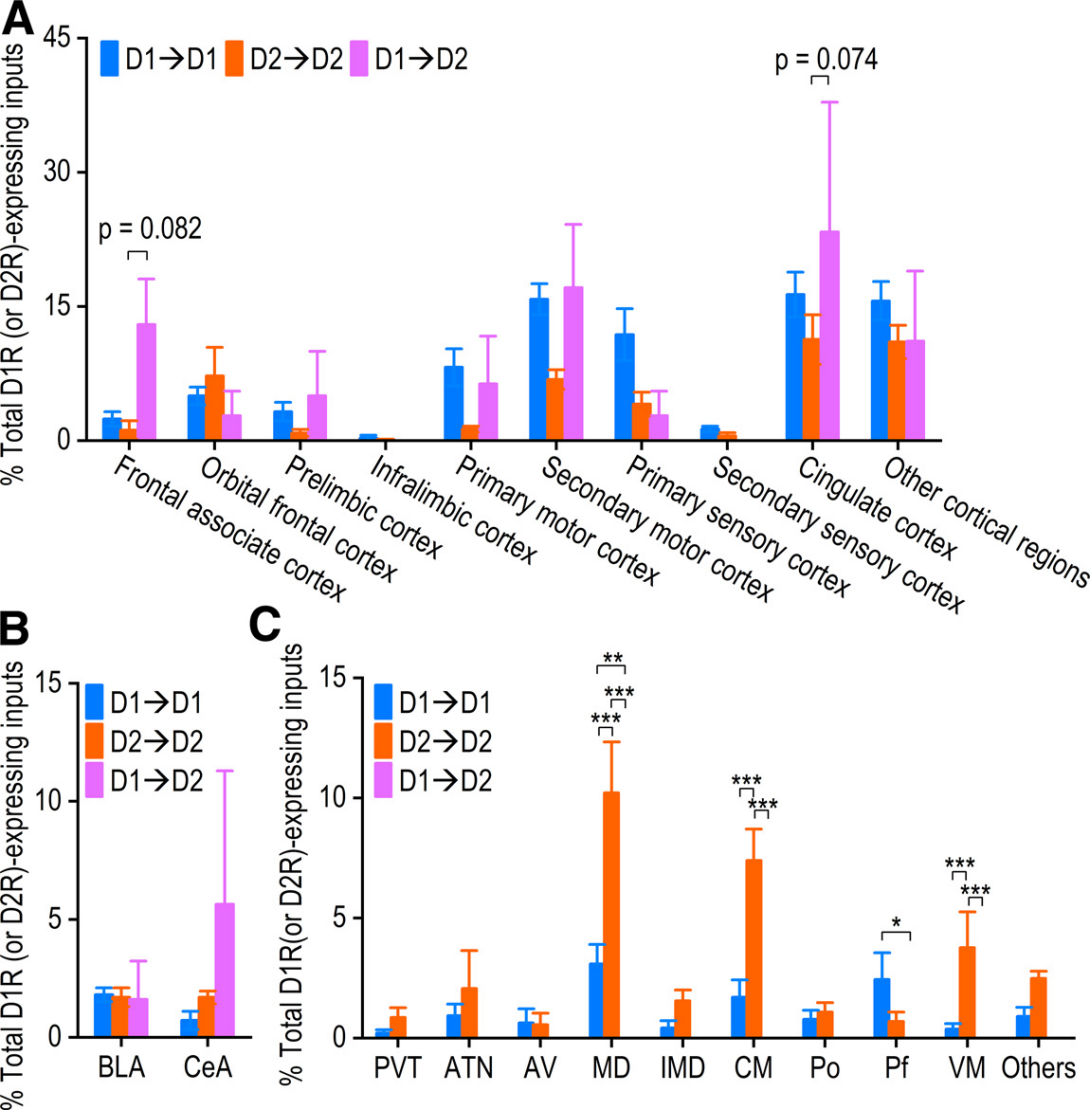
Distribution of extrastriatal D1R-expressing or D2R-expressing inputs onto MSNs from the cortex, amygdala, and thalamus. D1R-expressing or D2R-expressing inputs from the indicated brain regions were normalized to the total extrastriatal D1R-expressing or D2R-expressing inputs, as appropriate. ***A*,** Bar graph of cortical inputs, segregated into ten major subregions, indicating that the frontal associate cortex had a high proportion of D1→D2 connections; *p <* *0.05, two-way RM ANOVA followed by Tukey’s test. ***B*,** No significant differences were found in the proportions of connection types between the BLA or CeA and the pDMS. ***C*,** No significant differences were found in the proportions of connection types between the paraventricular thalamic nucleus (PVT), anterior thalamic nucleus (ATN), anteroventral thalamic nucleus (AV), intermediodorsal thalamic nucleus (IMD), or posterior thalamic nucleus (Po) and the pDMS. However, the mediodorsal thalamic nucleus (MD), central thalamic nucleus (CM), and ventromedial thalamic nucleus (VM) were found to have high percentages of D2→D2 connections, while the parafascicular thalamic nucleus (Pf) had a high percentage of D1→D1 connections; ***p <* *0.001, **p <* *0.01, *p <* *0.05, two-way RM ANOVA followed by Tukey’s test; *n* = 5 (D1→D1), *n* = 4 (D2→D2), *n* = 4 (D1→D2) mice.

We also analyzed the distribution of D1→D1, D2→D2, and D1→D2 in subregions of the cortex, amygdala, and thalamus that provided the most inputs to the pDMS. Analysis of ten cortical regions found a marginally higher percentage of D1→D2 than of D2→D2 projections from the frontal associate cortex (*p *=* *0.082; Fig. 5*A*). D1→D2 was also detected more frequently than D2→D2 from the cingulate cortex, although these differences did not achieve statistical significance (*p *=* *0.074; Fig. 5*A*). The basolateral amygdala and CeA contained similar percentages of D1→D1, D2→D2, and D1→D2 connections (*F*_(1,10)_ = 0.62, *p *>* *0.05; Fig. 5*B*). The data presented in Figure 5*C* showed that a significantly higher percentage of D2→D2 than of D1→D1 or D1→D2 projections arose from the mediodorsal thalamic nucleus (*p *<* *0.001), central thalamic nucleus (*p *<* *0.001), and ventromedial thalamic nucleus (*p *<* *0.001). A higher percentage of D1→D1 than of D1→D2 was observed in the parafascicular thalamic nucleus (*p *<* *0.05; Fig. 5*C*). Together, these results suggest that while the number of D1R-expressing or D2R-expressing neurons with projections to pDMS MSNs was low, they exhibited distinct brain distribution patterns.

## Discussion

Using the rabies virus-mediated retrograde monosynaptic tracing system, the present study investigated the inputs onto pDMS D1-MSNs and D2-MSNs and examined the distribution pattern of brain-wide extrastriatal D1R-expressing and D2R-expressing inputs to these MSNs. We found that the cortex, including the secondary motor and cingulate cortices, preferentially projects to pDMS D1-MSNs, whereas the thalamus, including the central and ventromedial thalamic nuclei, preferentially innervates pDMS D2-MSNs. In addition, while providing fewer inputs than the cortex and thalamus to the pDMS, the BNST and CeA contained more D2R-expressing than D1R-expressing neurons. Lastly, we discovered that MSN-projecting neurons exhibited distinct distribution patterns of the D1R and D2R across the whole brain. These results suggest that the segregation of connections between upstream brain regions and pDMS D1-MSNs or D2-MSNs may provide the basis for biased information propagation to basal ganglia structures, resulting in differential effects on behavior. In addition, D2R-expressing neurons in the BNST and CeA may exert distinct roles in anxiety and addiction.

### Distinct monosynaptic inputs to pDMS D1-MSNs and D2-MSNs

Given the broad roles of the striatum in motor execution, action selection, learning, behavioral flexibility, and reinforcement-associated behaviors such as reward-seeking and reinstatement (Ragozzino ME et al., 2002; Bamford et al., 2018; Roltsch Hellard et al., 2019), improving our understanding of how individual brain regions precisely innervate two major subtypes of striatal neurons represent an essential step toward dissecting the circuits that impact striatal function. The striatum extends a significant length along the anterior-posterior axis, and growing evidence (Yin and Knowlton, 2004; Yin et al., 2005a,b; Corbit and Janak, 2010; Shiflett et al., 2010; Regier et al., 2015) suggests a functional dissociation between anterior and posterior striatum. Although the coordinates that have been used for defining anterior and posterior of striatum varies, the coronal sections (e.g., −0.4 mm) posterior to the anterior commissure crossing the midline were usually considered as the posterior striatum in the majority of rat studies (Betancur et al., 1997; Yin and Knowlton, 2004; Yin et al., 2005a,b; Lex and Hauber, 2010; Shiflett et al., 2010; Stalnaker et al., 2010; Bradfield et al., 2013; Corbit et al., 2013; Hart and Balleine, 2016). According to the Paxinos atlas (Franklin and Paxinos, 2007), the anterior commissure crosses the midline more anteriorly in the mouse brain (at +0.14 mm) than in the rat brain (at −0.12 mm). Thus, a section at 0.0 mm from bregma in a mouse brain was used to define the posterior DMS (Shan et al., 2014; Matamales et al., 2016; Wang et al., 2018). Note that increasing evidence indicate that a third striatal portion along the anterior-posterior axis, the tail of the striatum, exhibits distinct functions and anatomic connections from aDMS or pDMS (Menegas et al., 2015, 2017, 2018; Matamales et al., 2016; Guo et al., 2018, 2019; Gangarossa et al., 2019).

The present study discovered that pDMS D1-MSNs and D2-MSNs received asymmetric inputs from other brain regions. We employed a monosynaptic rabies virus system (Wall et al., 2013) to label brain-wide neurons with direct connections to pDMS D1-MSNs or D2-MSNs. We discovered that these two MSN subtypes received asymmetric inputs from other whole brain regions. Dense D1-MSN innervations originated primarily from the orbital frontal, secondary motor, cingulate, and secondary visual cortices. These prefrontal and limbic structures are devoted mainly to reward motivation, emotional regulation, planning of complex cognitive behaviors, and decision-making (Floresco et al., 2008; Balleine et al., 2009; Gremel et al., 2016; Barthas and Kwan, 2017). Therefore, our results suggested that information related to reward, based on past experience, may be preferentially passed to the direct pathway circuit and thus facilitate actions likely to procure a reward. In contrast, D2-MSNs received inputs that mainly originated from the primary motor cortex, primary sensory cortex, central thalamic nucleus, and ventromedial thalamic nucleus. Concurrent activation of D1-MSNs and D2-MSNs has been reported during action initiation (Hikida et al., 2010; Cui et al., 2013). Biased projections from the primary motor and sensory cortices onto D2-MSNs may contribute to the suppression of unwanted behavior via activation of D2-MSNs. In addition, there is growing evidence that the thalamostriatal pathway plays an essential role in responses to salient stimuli and behavioral flexibility; projections from the central median and parafascicular nuclei onto cholinergic interneurons, which have also been found to express the D2R, appear to be particularly important in this context (Kreitzer and Malenka, 2008; Smith et al., 2011; Bradfield et al., 2013; Guo et al., 2015). The present study used D2-Cre mice to identify extrastriatal inputs onto D2-MSNs. Despite the relatively low abundance of striatal cholinergic interneurons (Kreitzer and Malenka, 2008), we cannot rule out the possibility that the observed connections between central and ventromedial thalamic regions and D2-expressing striatal neurons may also have included some projections to cholinergic interneurons. This architecture suggests that salient information from the thalamus may be differentially transmitted to D2R-expressing cholinergic interneurons and D2-MSNs to stop a current ongoing action and initiate a new action, thus facilitating flexible switching between behaviors.

The pDMS appears to be more involved in the acquisition of goal-directed actions than the aDMS (Yin et al., 2005a; Corbit and Janak, 2010; Shiflett et al., 2010), and the present study, therefore, focused on inputs to the pDMS. Our results were mostly consistent with other recent studies, which identified major inputs onto D1-MSNs and D2-MSNs that arose in the cortex and thalamus (Doig et al., 2010; Wall et al., 2013; Hunnicutt et al., 2016). Interestingly, in contrast to a previous observation (Wall et al., 2013), we identified significant projections from the secondary motor cortex and cingulate cortex to the pDMS. These inputs selectively innervated D1-MSNs. Anatomical and functional studies have suggested that the dorsomedial, dorsolateral, and ventral striatum received preferential inputs from the associative, sensorimotor, and limbic structures, respectively (Burton et al., 2015; Hunnicutt et al., 2016). Thus, this discrepancy may reflect the medial injection site employed in the present study.

### Distinct expression of D1Rs and D2Rs in the BNST and CeA

Interestingly, we observed that D2R-expressing neurons predominated in the BNST and CeA, which are closely associated with anxiety and addictive disorders (Davis et al., 2010; Kim et al., 2018). This was consistent with previous anatomic studies that identified a high proportion of D2R-expressing cells in the BNST and CeA using D2-GFP transgenic animals and D2 *in situ* hybridization (Perez de la Mora et al., 2012; De Bundel et al., 2016). The CeA and BNST receive dense dopaminergic inputs that originate in the ventral tegmental area (Hasue and Shammah-Lagnado, 2002; Krawczyk et al., 2011). A study using a mouse behavioral paradigm for auditory threat response generalization found that dopamine facilitated the consolidation of fear memory through concomitant activation of D2Rs in the CeA and BNST (De Bundel et al., 2016). A previous immunohistochemical study revealed that most D2R-expressing cells were also positive for protein kinase C-δ (Kim et al., 2018), a marker previously shown to identify GABAergic neurons. Selective expression of D2Rs in the BNST and CeA may have a critical impact on final behavioral outcomes in anxiety-related and reward-related processes.

### D1R-expressing or D2R-expressing inputs to D1-MSNs and D2-MSNs in the pDMS

Dopaminergic modulation of corticostriatal neurotransmission is particularly crucial during reward-based behaviors (Lüscher and Malenka, 2011; Bamford et al., 2018; Ma et al., 2018). A recent anatomic study observed high expression levels of D1Rs and D2Rs in the medial prefrontal cortex, orbital frontal cortex, and amygdala (Wei et al., 2018). Furthermore, these D1R-expressing and D2R-expressing neurons were highly segregated, with a low percentage of neurons co-expressing both D1Rs and D2Rs (Wei et al., 2018). Importantly, we found that D1R-expressing or D2R-expressing neurons within the cortex and amygdala sent projections to the DMS and formed functional connections with D1-MSNs or D2-MSNs (Lu et al., 2019). Thus, we expected that there would be strong anatomic connections between extrastriatal D1R-expressing or D2R-expressing DMS-projecting neurons and D1-MSNs or D2-MSNs. Surprisingly, we did not detect a high level of overlap between extrastriatal neurons expressing D1Rs or D2Rs and D1-MSNs or D2-MSNs; this suggested that the majority of extrastriatal neurons projecting to D1-MSNs or D2-MSNs did not express these receptors. Although only a minority of neurons with inputs to D1-MSNs or D2-MSNs expressed D1Rs or D2Rs, increasing evidence indicates that presynaptic D1Rs and D2Rs are essential for dopamine-dependent modulation of glutamate release at corticostriatal synapses (Wang and Goldman-Rakic, 2004; Bamford et al., 2018; Cui et al., 2018; Lu et al., 2019).

When the cortical inputs are stimulated at a low frequency, activation of presynaptic D1Rs was shown to boost glutamate release to D1 and D2-MSNs, whereas activation of presynaptic D2Rs suppressed glutamate release (Wang et al., 2012; Cui et al., 2018; Lu et al., 2019). However, when the cortical inputs are stimulated at a higher frequency, this dopaminergic modulation of glutamate release was blocked by adenosine and endocannabinoids (Bamford et al., 2018). This type of presynaptic dopaminergic modulation facilitates the appropriate selection and transmission of excitatory inputs via corticostriatal synapses during learning (Bamford et al., 2018). The D1R-expressing and D2R-expressing inputs to D1-MSNs or D2-MSNs may play important roles in this presynaptic filtration.

We observed that D1R-expressing or D2R-expressing neurons with inputs to pDMS D1-MSNs or D2-MSNs had distinct brain distribution patterns. Cortical regions contained a higher percentage of D1→D1 or D1→D2 connections, as compared with D2→D2, while thalamic areas had more D2→D2 connections, as compared with either D1→D1 or D1→D2. It is known that D1Rs exhibit a relatively low affinity for dopamine and are mainly activated by fast phasic release of high concentrations of dopamine, while D2Rs have a higher affinity for dopamine and mainly respond to slow tonic dopamine release (Richfield EK et al., 1989; Grieder et al., 2012). The relative levels of D1R-expressing and D2R-expressing inputs to MSNs from cortical and thalamic regions observed in this study suggest that the corticostriatal pathway may be more sensitive to phasic dopamine release. In contrast, the thalamostriatal pathway would be modulated by an increased basal dopamine level. These anatomic findings indicate that phasic dopamine release would preferentially initiate an action, while an increased basal dopamine level would stop an action. In addition, the SNc, lateral habenula, and BNST were similar to thalamic regions in that they expressed high levels of D2Rs than of D1Rs (Krawczyk et al., 2011; Ford, 2014). As we did not examine the anatomic connections between D2R-expressing inputs and D1-MSNs in the present study, it is not surprising that D2→D2 connections were more prevalent than either D1→D1 or D1→D2 in these regions.

Interestingly, the analysis of brain-wide inputs onto pDMS D1-MSNs and D2-MSNs revealed that orbital frontal cortex, secondary motor cortex, cingulate cortex, and secondary visual cortex preferentially innervated D1-MSNs, whereas the primary motor cortex, primary sensory cortex, central thalamic nucleus, ventromedial thalamic nucleus, and globus pallidus preferentially innervated D2-MSNs (Fig. 2). Besides geometric subdivisions, our study also included cell-type-specific inputs, based on their expression of D1Rs or D2Rs, onto either type of MSNs. We found that extrastriatal D1R-expressing or D2R-expressing inputs also showed biased projections to D1-MSNs or D2-MSNs in the pDMS (Fig. 5). It is worth noting that the subregion-based distinct input connectivity of D1-MSNs or D2-MSNs does not guarantee all types of neurons from this region show the same innervation preference. For instance, we did not find biased projection onto D1-MSNs versus D2-MSNs from the frontal associated cortex in Figure 2. However, we observed that D1R-expressing neurons in this brain region preferentially innervated D2-MSNs versus D1-MSNs in Figure 5. Thus, the innervation segregation of MSNs is not clear-cut based on the classification of brain structures. Therefore, it is of particular interest to understand how individual cell-type-specific inputs from different subregions precisely innervate the D1-MSNs and D2-MSNs.

### Technical consideration

The modified rabies virus-mediated monosynaptic tracing enables cell-specific tracing and has been a powerful tool to reveal brain circuitry. However, this method has some technical limitations. The variability of viral expression or imaging conditions may contribute to the discovered differences in the relative proportion of rabies-labeled input cells from certain brain areas onto either D1-MSNs or D2-MSNs. In addition, the efficiency of retrograde transmission of rabies virus at different excitatory, inhibitory, and neuromodulatory synapses is still not fully understood. We and others observed that the dopamine neurons innervating D1-MSNs or D2-MSNs only account for a small percentage of total neurons labeled by rabies virus (Wall et al., 2013; Smith et al., 2016), but the density of midbrain dopaminergic terminals and dopamine receptor expression were high within the striatum (Gerfen and Surmeier, 2011; Cheng et al., 2017; Wei et al., 2018). As compared with the corticostriatal excitatory synapses, those neuromodulatory synapses seem to be underestimated by rabies-mediated retrograde tracing. Similarly, the inhibitory synapses between BNST or CeA may be underreported by current data, as a strong inhibitory functional connection has been found from BNST to MSNs (Smith et al., 2016).

In summary, we have demonstrated that pDMS D1-MSNs and D2-MSNs received differential innervation from cortical and thalamic structures. Additionally, we found that the majority of brain-wide extrastriatal inputs to D1-MSNs or D2-MSNs did not express D1Rs or D2Rs, and that the input neurons that did express these receptors exhibited distinct distribution patterns. These findings provide a foundation for the understanding of information segregation in pDMS circuits that will guide future studies.

## Synthesis

Reviewing Editor: Karen Szumlinski, University of California at Santa Barbara Decisions are customarily a result of the Reviewing Editor and the peer reviewers coming together and discussing their recommendations until a consensus is reached. When revisions are invited, a fact-based synthesis statement explaining their decision and outlining what is needed to prepare a revision will be listed below. The following reviewer(s) agreed to reveal their identity: Yan Dong.

Based on the expert review of your report, the two reviewers and I have come to the decision of Revise and Re-review. This decision was based on a number of concerns regarding the relatively large number of caveats stemming from issues related to the methodology and data presentation. The specific issues of each reviewer are detailed below, but in particular, both reviewers and I request that the authors conduct a re-analysis of the existing data using more conventional statistical tests (i.e., no new data required). There is considerable emphasis on the region under study being localized to the posterior DMS and this is an issue of contention as the reviewers agree that the region studied in this paper is actually more anterior than that in the extant literature. Based on the images/sections available, the reviewers also agreed that a description of the starter site should be provided. There are a number of results and conclusions that are not directly supported by the data and both reviewers have listed specific concerns with suggestions on how to temper such conclusions. Finally, another common issue raised relates to the lack of a justification for inclusion of data pertaining to the BNST and CeA (Figures 4 and 5). We understand that we are requesting significant revision to this report but we agree that the revisions should require less than 2 months’ work, particularly given that no new data is required. However, given the pandemic, should you require an extension on the deadline for resubmission, please do not hesitate to contact eNeuro. Please see the individual reviewer comments below. You will note that Reviewer 1 expressed more concerns initially than Reviewer 2 but through Discussion the issues highlighted above (which were primary concerns of Reviewer 1) were agreed upon as being particularly critical to the revision.

Reviewer 1:

Overview: This study seeks to explore the brain-wide inputs to D1R+ and D2R+ MSNs in the mouse posterior DMS using a combination of viral and transgenic techniques. The authors use multiple Cre mouse lines and rabies virus combinations to demonstrate biases in innervation of the two MSN subclasses across multiple input regions, differential expression of D1Rs and D2Rs in the central amygdala and bed nucleus of the stria terminalis, and cell-type-specific differences in the expression of D1R and D2R in cells projecting to pDMS MSNs. While the study uses cutting-edge techniques and the question at hand is interesting, there are unfortunately several major flaws that detract from the report’s usefulness. The stated novelty of focusing on the posterior DMS rather than the anterior DMS as in previous studies (Wall et al. 2013) is unconvincing given that the coordinates and images of injection sites place the authors’ region in the anterior/middle aspect of the DMS rather than posterior. Identifying the dopamine receptor expression of the cells that project to the DMS has the potential to be quite interesting, but issues with the specific methodology used make the utility of these experiments limited. As such, I do not feel like the current manuscript advances our knowledge of striatal inputs in a meaningful way.

Statistics: hroughout the manuscript, the authors use the Student-Neuman-Keuls post-hoc test. This is not a commonly used statistical test. In fact, some outright caution against using it, as it is less conservative than the more commonly used and accepted tests, such as Tukey’s post hoc. As a result, it can show significant results that would not otherwise be seen with alternate tests. Due to the obscurity of the test, it would be impossible for a reader not well versed in this test to discern the stats in this manuscript. For example, I do not know how to properly interpret the q values listed throughout, and I imagine many others in our field would be confused as well. The statistics should be redone with a more accepted set of statistical analyses.

Other Comments:

1) Careful examination of the starter site is a vital part of a study that seeks to explore a very specific subregion, but this is impossible with the methodology used in much of the manuscript. Typically, when this set of viruses is used, starter neurons will be identified by colocalization of green and red fluorescence, resulting in yellow cells. Because fluorophores are used redundantly here (the starter virus and the rabies virus are both red), the extent of virus spread for both viruses is impossible to determine. This is an issue for a few reasons. For one, the accuracy of the injection site cannot be verified, making it hard to determine what is actually being traced in the experiment. There will always be mouse to mouse variability, so it’s essential to be able to analyze each mouse and throw out datasets that do not have an injection site entirely confined to the region of interest (which again is important if one is trying to distinguish these results from those obtained in another striatal subregion). Additionally, since there are both striatal and nonstriatal Cre+ neurons that project to the targeted Cre+ neurons in the DMS, it is possible that the terminals of these neurons could become infected by the starter virus and/or rabies virus. If this were to occur, rabies could potentially be labeling cells that are two synapses away from the cells of interest rather than one. Eliminating this possibility is impossible with the current use of fluorophores (or potentially some additional immunohistochemistry/immunofluorescence to differentiate the starter population). In later experiments, red fluorescence is present in both starter cells and in D1+ or D2+ cells, which again complicates interpretations. There is no benefit to using the mCherry-labeled starter virus rather than an unlabeled one (as used in Wall et al., 2013) if the starter population won’t be assessed, as it only adds an unnecessary and potentially confounding signal. Similarly, I don’t see the utility in using the EGFP double transgenic in the first set of experiments, and would have preferred to see the GFP rabies virus used instead.

2) The authors emphasize that they are focusing on the posterior DMS due to its specific behavioral roles, and state that this is unique from a previous DMS tracing paper which instead focused on the anterior portion of this region (Wall et al., 2012). However, the authors are injecting around 0.0 AP, and the images of the injection site clearly shows that this is not the posterior DMS. Their injection sites appear to lie in the anterior-middle portion of the DMS (though accurately assessing this for each mouse would not be possible, given the issues described in Major Point 1). In the Hunnicutt et al. study cited by the authors, the current injection site would not be considered to be in the posterior portion of the DMS. Given this, the novelty of the first set of results in this paper is limited.

3) Throughout the manuscript, the authors use the Student-Neuman-Keuls post-hoc test. This is not a commonly used statistical test. In fact, some outright caution against using it, as it is less conservative than the more commonly used and accepted tests, such as Tukey’s post hoc. As a result, it can show significant results that would not otherwise be seen with alternate tests. Due to the obscurity of the test, it would be impossible for a reader not well versed in this test to discern the stats in this manuscript. For example, I do not know how to properly interpret the q values listed throughout, and I imagine many others in our field would be confused as well. The statistics should be redone with a more accepted set of statistical analyses. As it is, I am skeptical of all statistical comparisons in the manuscript. Adding to the confusion is the fact that throughout the results section, it is frequently unclear what exactly is being compared, and the authors’ statements about the results do not always seem to line up with what could possibly be compared, given their methodology. For example, on line 327 they state that they observe “Significantly more D2->D2 connections than either D1->D1 or D1->D2 connections.” Given the lack of a starter population that can be normalized to, this statement isn’t clear to me. Is it the case that pDMS D2R MSN inputs are more D2R+ than inputs to D1R MSNs are? For this and many other statements, the result should be stated more clearly and should match the actual statistical comparison being made. Similarly, the statements in lines 212-215 are unsupported by the data - the authors can only describe the relative contribution of a given region to the overall inputs of an MSN subtype, but frequently make statements that overreach.

4) While the BNST and CeA D1R/D2R expression data are interesting, it is unclear what the relevance of this section of the manuscript is. These are not shown to be major inputs to the pDMS (by the authors or by others), so these anatomical results appear completely unrelated to the stated goals of the paper and should not be included here, unless there is a rationale that hasn’t been made clear. The authors also note in the Discussion that the predominance of D2Rs in these regions has already been observed with other methods, so it’s unclear what information the current data set provides.

Reviewer 2

This is a tight anatomical study aiming at understanding the potential biased innervation of pDMS D1 versus D2 neurons by presynaptic D1 versus D2 neurons. Reported findings provide solid information that enriches the overall D1-D2 segregation theory. The experimental designs are thorough, the approaches are solid, data appear to be solid, and conclusions are mostly reasonable. I only have several minor points, which may help the authors to improve the manuscript.

1) Data in figures 1 and 2 were obtained from D1 versus D2-cre lines separately, but direct comparison was made between groups. This can be troublesome because the variability of viral expression, imaging condition, etc. Ideally, such a comparison should be made within the same animals. It is understandable that this ideal scenario does not work in the authors’ current experimental setup, which use D1-cre and D2-cre lines. It is thus recommended to soften the conclusion as well as add discussions addressing this concern.

2) Data in figure 4 are potentially important. However, it may not be an optimal approach to pool extra-striatal neurons together, as it is known that brain regions that do not express dopamine receptors also project to the striatum. It would be helpful that, in addition to the summed results, the authors also provide the projection patterns (biased or unbiased) for a few key presynaptic regions. The authors should already have the data so it only need some additional data analysis.

3) Some of the image quality is not optimal. E.g., figure 4d the D1-to-D2 panel: it was not clear the red signals were cells or background or debris.

4) Discussion: Based on the data, it is reasonable to say that there is certain degree of segregation between D1 versus D2 neuron innervations. However, significant cross innervations were also observed. The data can also be used to argue that the innervation segregation is not clear-cut. Some discussions are welcome to address this alternative interpretation.

## Author Response

Dear Dr. Karen Szumlinski,

We greatly appreciate the editor and reviewers for their thoughtful comments. We have carefully revised the manuscript per the reviewers’ insightful suggestions and have provided our detailed responses below. The original concerns expressed by the reviewers are shown in italics.

Reviewer #1:

Overview: This study seeks to explore the brain-wide inputs to D1R+ and D2R+ MSNs in the mouse posterior DMS using a combination of viral and transgenic techniques. The authors use multiple Cre mouse lines and rabies virus combinations to demonstrate biases in innervation of the two MSN subclasses across multiple input regions, differential expression of D1Rs and D2Rs in the central amygdala and bed nucleus of the stria terminalis, and cell-type-specific differences in the expression of D1R and D2R in cells projecting to pDMS MSNs. While the study uses cutting-edge techniques and the question at hand is interesting, there are unfortunately several major flaws that detract from the report’s usefulness. The stated novelty of focusing on the posterior DMS rather than the anterior DMS as in previous studies (Wall et al. 2013) is unconvincing given that the coordinates and images of injection sites place the authors’ region in the anterior/middle aspect of the DMS rather than posterior. Identifying the dopamine receptor expression of the cells that project to the DMS has the potential to be quite interesting, but issues with the specific methodology used make the utility of these experiments limited. As such, I do not feel like the current manuscript advances our knowledge of striatal inputs in a meaningful way.

Response: We appreciate the reviewer’s comments and suggestions. Please see our responses below.

Statistics: throughout the manuscript, the authors use the Student-Neuman-Keuls post-hoc test. This is not a commonly used statistical test. In fact, some outright caution against using it, as it is less conservative than the more commonly used and accepted tests, such as Tukey’s post hoc. As a result, it can show significant results that would not otherwise be seen with alternate tests. Due to the obscurity of the test, it would be impossible for a reader not well versed in this test to discern the stats in this manuscript. For example, I do not know how to properly interpret the q values listed

throughout, and I imagine many others in our field would be confused as well. The statistics should be redone with a more accepted set of statistical analyses.

Response: As the reviewer suggested, we re-analyzed our data using the Tukey’s post-hoc test and found that 36 of 39 (92.31%) tests showed similar P values, leading to the same conclusions (p <

0.05) (Table 1 below). Nevertheless, we revised our manuscript using the Tukey test results for all data.

Other Comments:

1) Careful examination of the starter site is a vital part of a study that seeks to explore a very specific subregion, but this is impossible with the methodology used in much of the manuscript. Typically, when this set of viruses is used, starter neurons will be identified by colocalization of green and red

fluorescence, resulting in yellow cells. Because fluorophores are used redundantly here (the starter virus and the rabies virus are both red), the extent of virus spread for both viruses is impossible to determine. This is an issue for a few reasons. For one, the accuracy of the injection site cannot be verified, making it hard to determine what is actually being traced in the experiment. There will always be mouse to mouse variability, so it’s essential to be able to analyze each mouse and throw out datasets that do not have an injection site entirely confined to the region of interest (which again is important if one is trying to distinguish these results from those obtained in another striatal

subregion). Additionally, since there are both striatal and nonstriatal Cre+ neurons that project to the targeted Cre+ neurons in the DMS, it is possible that the terminals of these neurons could become infected by the starter virus and/or rabies virus. If this were to occur, rabies could potentially be labeling cells that are two synapses away from the cells of interest rather than one. Eliminating this possibility is impossible with the current use of fluorophores (or potentially some additional immunohistochemistry/immunofluorescence to differentiate the starter population). In later experiments, red fluorescence is present in both starter cells and in D1+ or D2+ cells, which again complicates interpretations. There is no benefit to using the mCherry-labeled starter virus rather than an unlabeled one (as used in Wall et al., 2013) if the starter population won’t be assessed, as it only adds an unnecessary and potentially confounding signal. Similarly, I don’t see the utility in using the EGFP double transgenic in the first set of experiments, and would have preferred to see the GFP rabies virus used instead.

Response: We appreciate the reviewer’s thoughtful comments and agree that because using GFP to identify the brain-wide D1R- or D2R-expressing neurons, we did not have an additional fluorescent color for the helper virus to label, together with the rabies virus, the starter cells. However, we believe that the identification of the starter cells is not required for making our conclusions for the following reasons. Both Wall et al. (2013, Figure 5G) and our data (Figures 1Diii and 1Dx) suggest that rabies injections caused an infection area of 0.75 mm in diameter. We injected the viruses at 0.0 mm posterior from the bregma; thus, the section containing the start of the injection site should be at

0.375 mm anterior from the bregma. Based on the pDMS coordinates in previous studies (Shan et al.,

2014; Matamales et al., 2016a; Matamales et al., 2016b) (Rebuttal Figure 1 below), we are still able to consider our rabies virus-infected region is within the pDMS. On the other hand, the researcher who conducted rabies infusions performed many rodent injections and implantations with very high precision. This reduced the off-target problem significantly.

Regarding the possibility that the rabies virus may label some neurons that were two synapses away from the D1-MSNs or D2-MSNs, we feel it unlikely occurred in our case because the helper virus we utilized in this study was Cre-dependent adeno-associated virus serotype 8 (AAV8)

expressing TVA and rabies glycoprotein. While some viruses (e.g., rabies) naturally exhibit retrograde transport, most AAV serotypes exhibit relatively low retrograde transport efficiency (Aschauer et al.,

2013; Tervo et al., 2016). Accumulating evidence suggested that the complete virus particles, including viral protein, were assembled in the cell body of neurons and then moved into axon and axon terminals (Tomishima and Enquist, 2001; Koyuncu et al., 2013). Therefore, although Cre- expressing axonal terminals might be infected by the helper virus, they still cannot express the TVA and rabies glycoprotein. TVA is an avian receptor protein that confers infection capability to pseudotyped rabies virus with the avian sarcoma leucosis virus glycoprotein EnvA (Wall et al., 2010; Wall et al., 2013). The rabies glycoprotein is necessary for mediating modified rabies monosynaptic retrograde spread (Wall et al., 2010; Wall et al., 2013). Lack of TVA and rabies glycoprotein, modified rabies virus cannot infect the Cre-expressing axonal terminals or spread retrogradely from its neurons.

In order to identify D1R- or D2R-expressing neurons across the brain, we utilized the double transgenic mice, D1-Cre;Snap25 and D2-Cre;Snap25 mice, which expressed GFP in Cre-expressing

cells. Given this, we need to use the rabies virus, which expressed mCherry, to distinguish neurons that project to the D1-MSNs or D2-MSNs. Due to the collateral connections of MSNs (Taverna et al.,

2008), no matter our helper virus with or without expressing mCherry, it is difficult to precisely analyze the number of starter cells. Similar approaches were used by Wall and colleagues. For instance, they utilized the GFP-expressing helper virus (AAV-EF1a-Flex-GTB) and mCherry-expressing rabies virus (EnvA-SAD-ΔG-mCherry) in D1-Cre and D2-Cre mice and reported that “our helper virus did not

allow for direct visualization of the number of starter cells” (Wall et al., 2013). As to the reason why

we did not utilize the single transgenic D1-Cre and D2-Cre for studying brain-wide inputs to D1-MSNs and D2-MSNs (Figs. 1 and 2), it is because we used the same animals as in Figures 4 and 5 to keep data consistency. In Figures 1 and 2 (whole-brain tracing of neurons that project to D1-MSNs and D2- MSNs), we analyzed rabies virus-infected red mCherry-expressing cells in extra-striatal brain regions. In Figures 4 and 5 (whole-brain tracing of D1R- or D2R-expressing neurons that project to D1-MSNs and D2-MSNs), we further analyzed whether they were mCherry-expressing cells in Figure 1 and 2 expressed GFP.

2) The authors emphasize that they are focusing on the posterior DMS due to its specific behavioral roles, and state that this is unique from a previous DMS tracing paper which instead focused on the anterior portion of this region (Wall et al., 2012). However, the authors are injecting around 0.0 AP, and the images of the injection site clearly shows that this is not the posterior DMS. Their injection sites appear to lie in the anterior-middle portion of the DMS (though accurately assessing this for each mouse would not be possible, given the issues described in Major Point 1). In the Hunnicutt et al. study cited by the authors, the current injection site would not be considered to be in the posterior portion of the DMS. Given this, the novelty of the first set of results in this paper is limited.

Response: We appreciate the reviewer’s comments. The reviewer brought up an important point regarding what is considered as the anterior DMS (aDMS) versus posterior DMS (pDMS).

pDMS was first reported in a behavioral study. The striatum extends a significant length along the anterior-posterior axis: 6.72 mm for rats (Paxinos and Watson, 2007) and 4.24 mm for mice (Franklin and Paxinos, 2007). In 1997, Betancur and colleagues reported that cocaine enhanced the expression of the neurotensin gene in neurons confined to the posterior dorsomedial striatum (AP:

0.4 mm posterior to bregma or -0.4 mm), but it did not alter this at the same region in the anterior striatum (AP: 1.2 mm anterior to bregma or +1.2 mm) in rats (Betancur et al., 1997). This is the first paper using the term of the posterior dorsomedial striatum. In 2004, Yin and Knowlton found that NMDA lesion of the posterior dorsomedial striatum or pDMS (AP: -0.4 mm), but not the anterior dorsomedial striatum or aDMS (AP: +1.2 mm), altered the rat spatially-guided behavior (Yin and Knowlton, 2004). In this paper, Yin et al. adapted the same AP coordinate as Betanucur et al. used and, for the first time, used pDMS and aDMS. Since then, a number of groups and publications reported the distinct roles of these two brain regions in rats (Betancur et al., 1997; Yin and Knowlton,

2004; Yin et al., 2005a; Yin et al., 2005b; Corbit and Janak, 2007; Pistell et al., 2009; Corbit and Janak, 2010; Lex and Hauber, 2010; Shiflett et al., 2010; Stalnaker et al., 2010; Bradfield et al., 2013; Corbit et al., 2013; Hart and Balleine, 2016; Bradfield and Balleine, 2017). For instance, Balleine’s group utilized the same coronal section (AP: -0.4 mm) as pDMS and provided evidence showing that the pDMS is crucial for the acquisition and expression of action-outcome association in instrumental learning (Yin et al., 2005a; Yin et al., 2005b). As shown in Table 2 below, most of the publication uses

-0.4 mm for pDMS and 1.2 mm for aDMS.

Landmark for the pDMS in the coronal section. According to the rat brain atlas by Paxinos and

Watson (Paxinos and Watson, 2007), a coronal section at an AP of -0.4 mm is just 2 sections (-0.24

mm, -0.36 mm) posterior to the anterior commissure (ac) crossing the midline (-0.12 mm) (Fig. 1A left below). A comparison of subregions between sections -0.24 and -0.36 mm found a new subregion, the third ventricle above the ac, appears at the section of 0.36 mm. This third ventricle above the ac can be used as an easily identified landmark for the pDMS section.

Mouse pDMS coordinates. However, a mouse brain is smaller than the rat one. Consequently, the mouse ac crosses the midline at +0.14 mm, which is more posterior than -0.12 mm in rats (Franklin and Paxinos, 2007) (Fig. 1 below). One section after the ac crossing the midline would be at

0.02 mm from bregma and this section starts to show the third ventricle above the ac. Thus, we considered that a mouse section at ∼0.02 mm is equivalent to a rat section at -0.4 mm that was used to define the posterior DMS. Indeed, as summarized in Table 2 below, several studies utilized coordinates centered around 0 mm from bregma as the anterior-posterior coordinates of posterior DMS (Shan et al., 2014; Matamales et al., 2016b; Wang et al., 2018). Thus, as discussed above, the injection site we used in the manuscript (AP: 0.0 mm) would be considered the posterior DMS (Figure

1C below).

Tail of the striatum. Increasing evidence recently indicate that a third striatal portion along the anterior-posterior axis, the tail of the striatum (TS), exhibits distinct functions and anatomic connections from aDMS or pDMS (Menegas et al., 2015; Matamales et al., 2016a; Menegas et al.,

2017; Guo et al., 2018; Menegas et al., 2018; Gangarossa et al., 2019; Guo et al., 2019). The TS ranges from -1.0 to -2.0. The TS is probably equivalent to the most posterior striatum reported in Hunnicutt et al. (Hunnicutt et al., 2016).

Wall and colleagues (Wall et al., 2013) injected rabies virus to the following coordinates: AP:

+0.32, ML: 2.00, DV: -3.12 for D1Cre mice; and AP: +0.48, ML: 2.04, DV: -3.04 for D2Cre mice. While these AP coordinates are between the aDMS and pDMS (or the beginning of the pDMS) (Rebuttal Figure 1B), their MLs and DVs make the injection sites are more dorsocentrally (Fig. 1C below) but

not dorsomedially (Fig. 1D below). Nevertheless, we soften the statement regarding the aDMS in the previous study in the revised manuscript. We also added the above points in the Discussion session.

Rebuttal Figure 1. A, Coronal atlas sections containing the posterior DMS in rats (left) and mice (right). Anterior commissure (ac) was highlighted in blue. 3V, third ventricle, highlighted in red. B, The coordinates (anterior-posterior) that were used for defining aDMS, pDMS, and TS in recent studies.

C, Injected sites used by Wall et al. (Figure 2F from Wall et al., 2013). D. Injection site in this study.

Adapted from manuscript Figure 1DX for comparison.

Table 2 Summary of coordinates used for aDMS, pDMS, and TS

3) Throughout the manuscript, the authors use the Student-Neuman-Keuls post-hoc test. This is not a commonly used statistical test. In fact, some outright caution against using it, as it is less conservative than the more commonly used and accepted tests, such as Tukey’s post hoc. As a result, it can show significant results that would not otherwise be seen with alternate tests. Due to the obscurity of the test, it would be impossible for a reader not well versed in this test to discern the stats in this manuscript. For example, I do not know how to properly interpret the q values listed throughout, and I imagine many others in our field would be confused as well. The statistics should be redone with a more accepted set of statistical analyses. As it is, I am skeptical of all statistical comparisons in the manuscript. Adding to the confusion is the fact that throughout the results section, it is frequently unclear what exactly is being compared, and the authors’ statements about the results do not always seem to line up with what could possibly be compared, given their methodology. For example, on line

327 they state that they observe “Significantly more D2->D2 connections than either D1->D1 or D1-

>D2 connections.” Given the lack of a starter population that can be normalized to, this statement isn’t clear to me. Is it the case that pDMS D2R MSN inputs are more D2R+ than inputs to D1R MSNs are? For this and many other statements, the result should be stated more clearly and should match the actual statistical comparison being made. Similarly, the statements in lines 212-215 are unsupported by the data - the authors can only describe the relative contribution of a given region to the overall inputs of an MSN subtype, but frequently make statements that overreach.

Response: We thank the reviewer for raising these concerns. Please see our response above to the

Statistics concern.

We apologize that we did not describe the results regarding Figure 4 and Figure 5 clearly in the Result session. On line 327 (line 357 now), we directly compared the number of D1R-expressing neurons that projected to the D1-MSNs (D1→D1), D2R-expressing neurons that projected to the D2- MSNs (D2→D2), and D1R-expressing neurons that projected the D2-MSNs (D1→D2). The D1→D1 was expressed as a percentage of the total number of the D1-MSN input, and D2→D2 or D1→D2 was expressed as a percentage of the D1-MSNs. Thus, in our comparison, we do not need the exact number of starter cells. So, we feel that a lack of access to the number of starter cells does not affect

our conclusion. After Figure 4E, we studied the distribution of the D1→D1, D1→D2, and D2→D2. The D1R- or D2R-expressing inputs from indicated brain regions, e.g., cortex and thalamus, were normalized to the total extra-striatal D1R- or D2R-expressing inputs, as appropriate. To prevent confusion, we added more description regarding corresponding comparisons and analysis in the Result session. We also soften the statement in line 212-215 (lines 238-241 now), as the reviewer suggested.

4) While the BNST and CeA D1R/D2R expression data are interesting, it is unclear what the relevance of this section of the manuscript is. These are not shown to be major inputs to the pDMS (by the authors or by others), so these anatomical results appear completely unrelated to the stated goals of the paper and should not be included here, unless there is a rationale that hasn’t been made clear. The authors also note in the Discussion that the predominance of D2Rs in these regions has already been observed with other methods, so it’s unclear what information the current data set provides.

Response: We thank the reviewer for raising this concern. We have now added the contents to address the rationale of why we studied the D1R or D2R expression in the BNST and CeA in the Result and Discussion session. The first reason we included the data regarding the D1R and D2R expression in BNST and CeA in this manuscript is that it enriches the map of D1R- and D2R- expressing neurons from extra-striatal regions that project to D1-MSNs and D2-MSNs. Dopamine signaling plays an important role in reward and addiction. D1Rs and D2Rs are the two major subtypes that mediate dopamine signaling. In addition to their postsynaptic expression in striatal MSNs, D1Rs and D2Rs are also present presynaptically at cortical terminals within the DMS. Importantly, a recent anatomical study revealed that the brain-wide DMS-projecting neurons preferentially express D2Rs versus D1Rs (Lu et al., 2019). Thus, having identified the distinct extra-

striatal inputs to D1-MSNs versus D2-MSNs (Figures 1 and 2), we next asked whether there was any difference in presynaptic inputs onto D1-MSNs or D2-MSNs in terms of their D1R and D2R expression, especially from the reward-associated brain areas that project to the DMS (e.g., cortex, amygdala, and BNST). Interestingly, although dopamine receptors are widely distributed in the brain, the receptor subtype densities vary between different areas. A previous study discovered that D1R-

or D2R-expressing neurons were highly segregated in the orbitofrontal cortex, prefrontal cortex, and basolateral amygdala (Wei et al., 2018). However, the brain regions that haven’t been reported are

the BNST and CeA, which are closely associated with anxiety and addictive disorders. Dopaminergic afferents from the ventral tegmental area innervate the amygdala and BNST, but the detailed D1R- and D2R-expression patterns remain unclear. While previous studies identified a high proportion of D2R-expressing cells in the BNST and CeA using D2-GFP transgenic animals and D2 in situ hybridization (Perez de la Mora et al., 2012; De Bundel et al., 2016), but our triple transgenic mouse model directly demonstrated that D2R-expressing neurons were significantly more than those of the D1R-expressing one (Figure 3). In addition, the extra-striatal D1R- or D2R-expressing neurons formed functional connections with D1-MSNs or D2-MSNs (Lu et al., 2019). Therefore, we lastly investigated the anatomical connections between extra-striatal D1R- or D2R-expressing DMS- projecting neurons and D1-MSNs or D2-MSNs (Figures 4 and 5).

The second reason we studied the dopamine receptor expression pattern in the BNST and CeA is that these two brain regions may still be one of the major inputs to pDMS, even though it does not support by our data presented here (but see Smith et al., 2016). The efficiency of retrograde transmission of rabies virus at different excitatory, inhibitory, and neuromodulatory synapses still remains incompletely known. We and others observed innervation of midbrain dopamine onto D1- MSNs or D2-MSNs only account for a small percentage of total neuronal labeled by rabies virus (Wall et al., 2013; Smith et al., 2016), but the density of midbrain dopaminergic terminals and dopamine receptor expression were extensive high in the striatum (Gerfen and Surmeier, 2011; Cheng et al.,

2017; Wei et al., 2018). As compared to the corticostriatal excitatory synapses, those neuromodulatory synapses seem to be underestimated by rabies-mediated retrograde tracing. Similarly, the inhibitory synapses between BNST or CeA may be underreported by current data, as a strong inhibitory functional connection has been found from BNST to MSNs (Smith et al., 2016).

Reviewer #2:

This is a tight anatomical study aiming at understanding the potential biased innervation of pDMS D1 versus D2 neurons by presynaptic D1 versus D2 neurons. Reported findings provide solid information that enriches the overall D1-D2 segregation theory. The experimental designs are thorough, the approaches are solid, data appear to be solid, and conclusions are mostly reasonable. I only have several minor points, which may help the authors to improve the manuscript.

1) Data in figures 1 and 2 were obtained from D1 versus D2-cre lines separately, but direct comparison was made between groups. This can be troublesome because the variability of viral expression, imaging condition, etc. Ideally, such a comparison should be made within the same animals. It is understandable that this ideal scenario does not work in the authors’ current experimental setup, which use D1-cre and D2-cre lines. It is thus recommended to soften the conclusion as well as add discussions addressing this concern.

Response: We agree with the reviewer that the variability of vial expression or imaging condition may contribute to the discovered differences in the relative proportion of rabies-labeled inputs cells from certain brain areas onto either D1-MSNs or D2-MSNs. The clarification relating to this aspect of the study has been added to the Discussion in the revised manuscript. However, to compensate for the effect of the individual variation in Figures 1 and 2, we did not directly compare the absolute number

of rabies-labeled cells between D1-Cre and D2-Cre lines from a given brain area. Since there was no significant difference in the total number of rabies-labeled neurons between D1-Cre and D2-Cre lines, we normalized the number of rabies-labeled cells in each area by the total number of rabies-labeled neurons in each brain. Thus, variations of viral infusion, viral expression, and imaging condition are

less likely to affect the comparison in normalized distributions of rabies-labeled across different brain areas between D1-Cre and D2-Cre lines.

2) Data in figure 4 are potentially important. However, it may not be an optimal approach to pool extra-striatal neurons together, as it is known that brain regions that do not express dopamine receptors also project to the striatum. It would be helpful that, in addition to the summed results, the authors also provide the projection patterns (biased or unbiased) for a few key presynaptic regions. The authors should already have the data so it only need some additional data analysis.

Response: We appreciate the reviewer’s suggestions. We analyzed the distribution of D1→D1, D2→D2, and D1→D2 in sub-regions of the cortex and thalamus that provided the most inputs to the D1-MSNs and D2-MSNs in Figure 5, including the key presynaptic regions to pDMS, i.e., orbital frontal cortex, prelimbic cortex, motor cortex, and sensory cortex.

3) Some of the image quality is not optimal. E.g., figure 4d the D1-to-D2 panel: it was not clear the red signals were cells or background or debris.

Response: We apologize for the unclear image. We now changed the image for D1→D2 panel in

Figure 4D, as the reviewer indicated.

4) Discussion: Based on the data, it is reasonable to say that there is certain degree of segregation between D1 versus D2 neuron innervations. However, significant cross innervations were also observed. The data can also be used to argue that the innervation segregation is not clear-cut. Some discussions are welcome to address this alternative interpretation.

Response: We agree with the reviewer and greatly appreciate this suggestion. The analysis of brain- wide input onto pDMS D1-MSNs and D2-MSNs revealed that orbital frontal cortex, secondary motor cortex, cingulate cortex, and secondary visual cortex preferentially innervated D1-MSNs, whereas the primary motor cortex, primary sensory cortex, central thalamic nucleus, ventromedial thalamic nucleus, and globus pallidus preferentially innervated D2-MSNs (Figure 2). Besides geometric subdivisions, our study also includes cell-type-specific inputs onto either type of MSNs based on dopamine receptors, i.e., D1R- or D2R-expression inputs. We found that extra-striatal D1R- or D2R-

expressing inputs also showed biased projections to D1-MSNs or D2-MSNs in the pDMS (Figure 5). It is worth noting that the subregion-based distinct input connectivity of D1-MSNs or D2-MSNs does not guarantee all types of neurons from this region show the same innervation preference. For instance, we did not find biased projection onto D1-MSNs versus D2-MSNs from the frontal associated cortex

in Figure 2. However, we observed that D1R-expressing neurons in this brain region preferentially innervated D2-MSNs versus D1-MSNs in Figure 5. Thus, the innervation segregation of MSNs is not clear-cut based on the classification of brain structures. Therefore, it is of particular interest in understanding how individual cell-type-specific inputs from different subregions precisely innervation the D1-MSNs and D2-MSNs. We have added the above points in our Discussion.

Other changes:

All changes in the text use a blue font

REFERENCES

Aschauer DF, Kreuz S, Rumpel S (2013) Analysis of transduction efficiency, tropism and axonal transport of AAV serotypes 1, 2, 5, 6, 8 and 9 in the mouse brain. PLoS One 8:e76310.

Betancur C, Rostene W, Berod A (1997) Chronic cocaine increases neurotensin gene expression in the shell of the nucleus accumbens and in discrete regions of the striatum. Brain Res Mol Brain Res 44:334-340.

Bradfield LA, Balleine BW (2017) Thalamic control of dorsomedial striatum regulates internal state to guide goal-directed action selection. J Neurosci 37:3721-3733.

Bradfield LA, Bertran-Gonzalez J, Chieng B, Balleine BW (2013) The thalamostriatal pathway and cholinergic control of goal-directed action: interlacing new with existing learning in the striatum. Neuron 79:153-166.

Cheng Y, Huang CC, Ma T, Wei X, Wang X, Lu J, Wang J (2017) Distinct synaptic strengthening of the striatal direct and indirect pathways drives alcohol consumption. Biol Psychiatry 81:918-

929.

Corbit LH, Janak PH (2007) Inactivation of the lateral but not medial dorsal striatum eliminates the excitatory impact of Pavlovian stimuli on instrumental responding. J Neurosci 27:13977-13981.

Corbit LH, Janak PH (2010) Posterior dorsomedial striatum is critical for both selective instrumental and Pavlovian reward learning. Eur J Neurosci 31:1312-1321.

Corbit LH, Leung BK, Balleine BW (2013) The role of the amygdala-striatal pathway in the acquisition and performance of goal-directed instrumental actions. J Neurosci 33:17682-17690.

De Bundel D, Zussy C, Espallergues J, Gerfen CR, Girault JA, Valjent E (2016) Dopamine D2 receptors gate generalization of conditioned threat responses through mTORC1 signaling in the extended amygdala. Mol Psychiatry 21:1545-1553.

Franklin KBJ, Paxinos G (2007) The mouse brain in stereotaxic coordinates, 3 Edition. San Diego: Academic Press.

Gangarossa G, Castell L, Castro L, Tarot P, Veyrunes F, Vincent P, Bertaso F, Valjent E (2019) Contrasting patterns of ERK activation in the tail of the striatum in response to aversive and rewarding signals. J Neurochem 151:204-226.

Gerfen CR, Surmeier DJ (2011) Modulation of striatal projection systems by dopamine. Annu Rev

Neurosci 34:441-466.

Guo L, Walker WI, Ponvert ND, Penix PL, Jaramillo S (2018) Stable representation of sounds in the posterior striatum during flexible auditory decisions. Nat Commun 9:1534.

Guo L, Weems JT, Walker WI, Levichev A, Jaramillo S (2019) Choice-Selective Neurons in the

Auditory Cortex and in Its Striatal Target Encode Reward Expectation. J Neurosci 39:3687-

3697.

Hart G, Balleine BW (2016) Consolidation of Goal-Directed Action Depends on MAPK/ERK Signaling in Rodent Prelimbic Cortex. J Neurosci 36:11974-11986.

Hunnicutt BJ, Jongbloets BC, Birdsong WT, Gertz KJ, Zhong H, Mao T (2016) A comprehensive excitatory input map of the striatum reveals novel functional organization. Elife 5:e19103.

Koyuncu OO, Hogue IB, Enquist LW (2013) Virus infections in the nervous system. Cell Host Microbe

13:379-393.

Lee CR, Yonk AJ, Wiskerke J, Paradiso KG, Tepper JM, Margolis DJ (2019) Opposing influence of sensory and motor cortical input on striatal circuitry and choice behavior. Curr Biol 29:1313-

1323.

Lex B, Hauber W (2010) Disconnection of the entorhinal cortex and dorsomedial striatum impairs the sensitivity to instrumental contingency degradation. Neuropsychopharmacology 35:1788-1796.

Lu J, Cheng Y, Wang X, Woodson K, Kemper C, Disney E, Wang J (2019) Alcohol intake enhances glutamatergic transmission from D2 receptor-expressing afferents onto D1 receptor-expressing medium spiny neurons in the dorsomedial striatum. Neuropsychopharmacology 44:1123-1131.

Matamales M, Gotz J, Bertran-Gonzalez J (2016a) Quantitative Imaging of Cholinergic Interneurons Reveals a Distinctive Spatial Organization and a Functional Gradient across the Mouse Striatum. PLoS One 11:e0157682.

Matamales M, Skrbis Z, Hatch RJ, Balleine BW, Gotz J, Bertran-Gonzalez J (2016b) Aging-related dysfunction of striatal cholinergic interneurons produces conflict in action selection. Neuron

90:362-373.

Menegas W, Babayan BM, Uchida N, Watabe-Uchida M (2017) Opposite initialization to novel cues in dopamine signaling in ventral and posterior striatum in mice. Elife 6.

Menegas W, Akiti K, Amo R, Uchida N, Watabe-Uchida M (2018) Dopamine neurons projecting to the posterior striatum reinforce avoidance of threatening stimuli. Nat Neurosci 21:1421-1430.

Menegas W, Bergan JF, Ogawa SK, Isogai Y, Umadevi Venkataraju K, Osten P, Uchida N, Watabe- Uchida M (2015) Dopamine neurons projecting to the posterior striatum form an anatomically distinct subclass. Elife 4:e10032.

Paxinos G, Watson C (2007) The Rat Brain in stereotaxic coordinates, 6 Edition. San Diego: Academic Press.

Perez de la Mora M, Gallegos-Cari A, Crespo-Ramirez M, Marcellino D, Hansson AC, Fuxe K (2012) Distribution of dopamine D(2)-like receptors in the rat amygdala and their role in the modulation of unconditioned fear and anxiety. Neuroscience 201:252-266.

Pistell PJ, Nelson CM, Miller MG, Spangler EL, Ingram DK, Devan BD (2009) Striatal lesions interfere with acquisition of a complex maze task in rats. Behav Brain Res 197:138-143.

Regier PS, Amemiya S, Redish AD (2015) Hippocampus and subregions of the dorsal striatum respond differently to a behavioral strategy change on a spatial navigation task. J Neurophysiol

114:1399-1416.

Shan Q, Ge M, Christie MJ, Balleine BW (2014) The acquisition of goal-directed actions generates opposing plasticity in direct and indirect pathways in dorsomedial striatum. J Neurosci

34:9196-9201.

Shiflett MW, Brown RA, Balleine BW (2010) Acquisition and performance of goal-directed instrumental actions depends on ERK signaling in distinct regions of dorsal striatum in rats. J Neurosci 30:2951-2959.

Smith JB, Klug JR, Ross DL, Howard CD, Hollon NG, Ko VI, Hoffman H, Callaway EM, Gerfen CR, Jin X (2016) Genetic-based dissection unveils the inputs and outputs of striatal patch and matrix compartments. Neuron 91:1069-1084.

Stalnaker TA, Calhoon GG, Ogawa M, Roesch MR, Schoenbaum G (2010) Neural correlates of stimulus-response and response-outcome associations in dorsolateral versus dorsomedial striatum. Front Integr Neurosci 4:12.

Taverna S, Ilijic E, Surmeier DJ (2008) Recurrent collateral connections of striatal medium spiny neurons are disrupted in models of Parkinson’s disease. J Neurosci 28:5504-5512.

Tervo DG, Hwang BY, Viswanathan S, Gaj T, Lavzin M, Ritola KD, Lindo S, Michael S, Kuleshova E, Ojala D, Huang CC, Gerfen CR, Schiller J, Dudman JT, Hantman AW, Looger LL, Schaffer DV, Karpova AY (2016) A Designer AAV Variant Permits Efficient Retrograde Access to Projection Neurons. Neuron 92:372-382.

Tomishima MJ, Enquist LW (2001) A conserved alpha-herpesvirus protein necessary for axonal localization of viral membrane proteins. J Cell Biol 154:741-752.

Wall NR, De La Parra M, Callaway EM, Kreitzer AC (2013) Differential innervation of direct- and indirect-pathway striatal projection neurons. Neuron 79:347-360.

Wall NR, Wickersham IR, Cetin A, De La Parra M, Callaway EM (2010) Monosynaptic circuit tracing in vivo through Cre-dependent targeting and complementation of modified rabies virus. Proc Natl Acad Sci U S A 107:21848-21853.

Wang L, Rangarajan KV, Gerfen CR, Krauzlis RJ (2018) Activation of Striatal Neurons Causes a Perceptual Decision Bias during Visual Change Detection in Mice. Neuron 97:1369-1381 e1365.

Wei X, Ma T, Cheng Y, Huang CCY, Wang X, Lu J, Wang J (2018) Dopamine D1 or D2 receptor- expressing neurons in the central nervous system. Addiction biology 23:569-584.

Yin HH, Knowlton BJ (2004) Contributions of striatal subregions to place and response learning.

Learn Mem 11:459-463.

Yin HH, Knowlton BJ, Balleine BW (2005a) Blockade of NMDA receptors in the dorsomedial striatum prevents action-outcome learning in instrumental conditioning. Eur J Neurosci 22:505-512.

Yin HH, Ostlund SB, Knowlton BJ, Balleine BW (2005b) The role of the dorsomedial striatum in instrumental conditioning. Eur J Neurosci 22:513-523.
